# The spleen microenvironment influences disease transformation in a mouse model of KIT^D816V^-dependent myeloproliferative neoplasm

**DOI:** 10.1038/srep41427

**Published:** 2017-01-27

**Authors:** Natalie Pelusi, Maike Kosanke, Tamara Riedt, Corinna Rösseler, Kristin Seré, Jin Li, Ines Gütgemann, Martin Zenke, Viktor Janzen, Hubert Schorle

**Affiliations:** 1 University of Bonn Medical School, Institute of Pathology, Department of Developmental Pathology, 53127 Bonn, Germany; 2 Institute for Biomedical Engineering, Department of Cell Biology, RWTH Aachen University Medical School, Pauwelsstr. 30, 52074 Aachen, Germany; 3 Helmholtz Institute for Biomedical Engineering, RWTH Aachen University, Pauwelsstr. 20, 52074 Aachen, Germany; 4 University of Bonn Medical School, Department of Internal Medicine III, Hematology and Oncology, Sigmund-Freud-Str. 25, 53127 Bonn, Germany

## Abstract

Activating mutations leading to ligand-independent signaling of the stem cell factor receptor KIT are associated with several hematopoietic malignancies. One of the most common alterations is the D816V mutation. In this study, we characterized mice, which conditionally express the humanized KIT^D816V^ receptor in the adult hematopoietic system to determine the pathological consequences of unrestrained KIT signaling during blood cell development. We found that KIT^D816V^ mutant animals acquired a myeloproliferative neoplasm similar to polycythemia vera, marked by a massive increase in red blood cells and severe splenomegaly caused by excessive extramedullary erythropoiesis. Moreover, we found mobilization of stem cells from bone marrow to the spleen. Splenectomy prior to KIT^D816V^ induction prevented expansion of red blood cells, but rapidly lead to a state of aplastic anemia and bone marrow fibrosis, reminiscent of post polycythemic myeloid metaplasia, the spent phase of polycythemia vera. Our results show that the extramedullary hematopoietic niche microenvironment significantly influences disease outcome in KIT^D816V^ mutant mice, turning this model a valuable tool for studying the interplay between functionally abnormal hematopoietic cells and their microenvironment during development of polycythemia vera-like disease and myelofibrosis.

The blood forming system is characterized by a remarkable regenerative capacity, required for the continuous replacement of mature blood cells. During that process, the balance between cell proliferation and differentiation has to be tightly controlled to prevent hematopoietic malignancies. The KIT receptor, expressed by hematopoietic stem cells (HSCs) and progenitor cells (HPCs) and several lineage-committed precursors, is involved in cell maintenance, proliferation, survival and terminal differentiation^1^,^2^,^3^,^4^,^5^,^6^. Uncontrolled KIT signaling is associated with several myeloproliferative disorders^7^,^8^,^9^.

KIT belongs to the type III subfamily of tyrosine kinases and gets activated by its ligand stem cell factor (SCF), which is expressed as a membrane bound or soluble form^10^,^11^,^12^. SCF is produced by stromal cells in the hematopoietic bone marrow (BM) niche. For HSCs, interaction of KIT with membrane bound SCF was shown to be important for positioning to the niche^13^,^14^. Furthermore, KIT was described to be important for the maintenance of long-term HSCs^5^. In most lineages, KIT is downregulated during differentiation^2^, while in mast cells high KIT expression is maintained^15^. KIT deficient mice die in utero due to defects in fetal liver erythropoiesis, demonstrating its important function in red blood cells^16^. In erythroid progenitors, KIT regulates proliferation and maintenance of the undifferentiated state^17^.

Several KIT mutations have been described that cause constitutive receptor activation without ligand binding. The D816V substitution is one of the most commonly described mutations associated with hematopoietic neoplasia^8^,^9^,^18^. We previously described the generation of a humanized transgenic mouse model for conditional KIT^D816V^ expression and analyzed effects of KIT^D816V^ signaling on fetal liver erythropoiesis^19^. Here, we used R26-LSL-KIT^D816V^ mice to investigate sustained KIT^D816V^ signaling in the adult hematopoietic system and found development of a myeloproliferative neoplasm (MPN) reminiscent of polycythemia vera (PV), which was transplantable and characterized by massively increased red cell mass and splenomegaly. Furthermore, stem cells were mobilized from BM to the spleen. Splenectomy of KIT^D816V^ mutants prevented the increase in red cell mass but promoted BM failure and myelofibrosis, clinical features observed upon transformation of PV to post polycythemic myeloid metaplasia. The fact that course of disease in KIT^D816V^ mutants is influenced by splenectomy demonstrates the relevance of the niche and provides a unique model to study the interdependency of hematopoietic cells and the microenvironment.

## Results

### KIT^D816V^ induces a polycythemia vera-like disease

**Figure 1:**
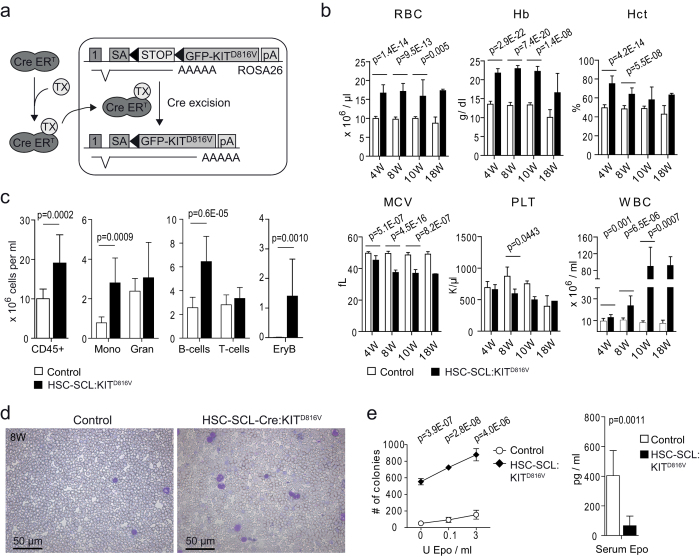
Peripheral blood analysis reveals red blood cell and white blood cell hyperplasia reminiscent of polycythemia vera in HSC-SCL:KIT^D816V^ mice. (**a**) Schematic illustration of tamoxifen (TX)-dependent KIT^D816V^ transgene induction in HSC-SCL:KIT^D816V^ mice. A *loxP*-flanked transcriptional STOP cassette precedes the the KIT^D816V^ cDNA that is fused to a GFP (*pontellina plumata*) via a 2A-peptide. The Cre-ER^T^ protein is expressed in hematopoietic cells and translocates to the nucleus upon TX binding. Thereafter, GFP-KIT^D816V^ expression is driven by the endogenous *ROSA26* promoter, mediating moderate transgene expression^64^,^65^. 1: *ROSA26* Exon1. pA: polyadenylation sequence. SA: splice acceptor. (**b**) Indicated pB parameters of HSC-SCL:KIT^D816V^ and control mice were analyzed 4, 8, 10 and 18 weeks after TX injection with a hematology analyzer. 4 W - Control: N = 20. HSC-SCL:KIT^D816V^: N = 14. 8 W - Control: N = 17. HSC-SCL:KIT^D816V^: N = 9. 10 W - Control: N = 7. HSC-SCL:KIT^D816V^: N = 6. 18 W - Control: N = 4. HSC-SCL:KIT^D816V^: N = 2. (**c**) 8 weeks after TX injection, pB from HSC-SCL:KIT^D816V^ and control mice was subjected to erythrocyte lysis and nucleated cells were analyzed for expression of CD45 and markers for lineage committed blood cells via flow cytometry. The WBC was used to calculate the absolute number of cells per unit of blood volume. Control: N = 12. HSC-SCL:KIT^D816V^: N = 7. EryB: Erythroblasts. Gran: Granulocytes. Mono: Monocytes. (**d**) Blood smears of HSC-SCL:KIT^D816V^ and control mice were prepared 8 weeks after TX injection and subjected to May-Grünwald-Giemsa staining. (**e**) Left: Number of splenic CFU-E colonies obtained at indicated concentrations of supplemented erythropoietin. As MethoCult methylcellulose medium includes fetal bovine serum as a possible source of erythropoietin, data demonstrate hyper-responsiveness of HSC-SCL:KIT^D816V^ CFU-Es to erythropoietin but cannot prove full erythropoietin-independency. Analysis carried out 10 weeks after induction. Control: N = 4. HSC-SCL:KIT^D816V^: N = 4. Right: Serum erythropoietin levels were determined 8 weeks after induction using ELISA. Control: N = 6. HSC-SCL:KIT^D816V^: N = 6. Data are presented as mean ± standard deviation. P-values were determined using two-tailed, unpaired Student’s t-test.

We previously described the generation of the R26-LSL-KIT^D816V^ mouse line, harboring a conditional knock in of a humanized KIT^D816V^ receptor linked to a green fluorescent protein (GFP) in the *ROSA26* genomic locus. The D816V mutation has been implicated in the pathology of acute myeloid leukemia, mastocytosis and other oncogenic malignancies^7^,^8^,^9^,^18^,^20^. To extend the knowledge on how KIT regulates hematopoiesis and contributes to myeloproliferative disorders, we studied the effects of ectopic KIT^D816V^ expression in the adult hematopoietic system. We mated R26-LSL-KIT^D816V^ with HSC-SCL-Cre-ER^T^ mice, which express a tamoxifen-inducible Cre recombinase under control of the stem cell enhancer of the *Scl* gene locus^21^. HSC-SCL-Cre-ER^T^-mediated recombination has been demonstrated in HSCs/HPCs and endothelial cells. Double transgenic HSC-SCL-Cre-ER^T^:R26-LSL-KIT^D816V^ animals (hereafter called HSC-SCL:KIT^D816V^) were viable and developed normally. For induction of KIT^D816V^ expression (Fig. 1a), we treated adult HSC-SCL:KIT^D816V^ mice with a daily dose of 1.5 mg tamoxifen (TX) for 5 consecutive days. TX-treated wildtype and single transgenic littermates were used as controls. Quantitative real-time PCR validated KIT^D816V^ expression in hematopoietic compartments of HSC-SCL:KIT^D816V^ animals after induction, with transcript levels comparable to endogenous Kit expression in controls. To validate GFP co-expression, GFP-positive and -negative fractions were also analyzed for KIT^D816V^ expression (Supplementary Fig. S1).

In total, 44 HSC-SCL:KIT^D816V^ and 45 control mice were monitored for 4–10 weeks after treatment. Only a limited group of 2 HSC-SCL:KIT^D816V^ and 4 control animals was monitored up to 18 weeks, as we observed a high rate of spontaneous mortality for HSC-SCL:KIT^D816V^ animals (29.55%; 13/44 mice) within the first 10 weeks after induction. Aside from moderate enlargement of the abdomen in some cases, HSC-SCL:KIT^D816V^ mice showed no signs of morbidity before death. From the control group, all animals survived the observation period.

Cell counts in peripheral blood (pB) were analyzed 4, 8, 10 and 18 weeks after induction. The red blood cell count (RBC), hemoglobin (Hb), mean platelet volume (MPV) and white blood cell count (WBC) were significantly elevated in HSC-SCL:KIT^D816V^ mice compared to controls (Fig. 1b, Supplementary Fig. S2). The hematocrit (Hct) was initially elevated to high levels and slightly decreased thereafter. The mean corpuscular volume (MCV) and the platelet count (PLT) were decreased in HSC-SCL:KIT^D816V^ mice.

We further investigated alterations in pB cell lineages via flow cytometry. Analyses demonstrated elevated amounts of CD45-positive cells in pB of HSC-SCL:KIT^D816V^ mice, caused by increased monocyte and B-cell populations and a mild increase in granulocytes. Staining for erythroid markers CD71 and Ter-119^22^ revealed the presence of CD45-negative erythroblasts, which are normally not released into circulation (Fig. 1c, Supplementary Fig. S2). In addition, the reticulocyte frequency was increased (Supplementary Fig. S2). Figure 1d shows pB smears of HSC-SCL:KIT^D816V^ and control mice 8 weeks after induction. Accumulation of erythrocytes and mature myeloid cells is a hallmark of PV^23^. PV furthermore often includes thrombocytosis, but similar to murine PV models harboring the JAK2^V617F^ mutation^24^,^25^,^26^ we found no elevation of platelets in HSC-SCL:KIT^D816V^ mice. As PV is additionally marked by endogenous erythroid colony formation and decreased serum erythropoietin^23^, we further analyzed these parameters. Indeed, for HSC-SCL:KIT^D816V^ mice markedly decreased serum erythropoietin levels and high numbers of splenic colony forming unit-erythroids (CFU-Es) in assays with low erythropoietin concentration were found, indicating erythropoietin hyper-responsiveness (Fig. 1d). Thrombopoietin serum levels were also reduced in HSC-SCL:KIT^D816V^ mice (Supplementary Fig. S2).

### HSC-SCL:KIT^D816V^ mice develop splenomegaly with massive extramedullary erythropoiesis

**Figure 2:**
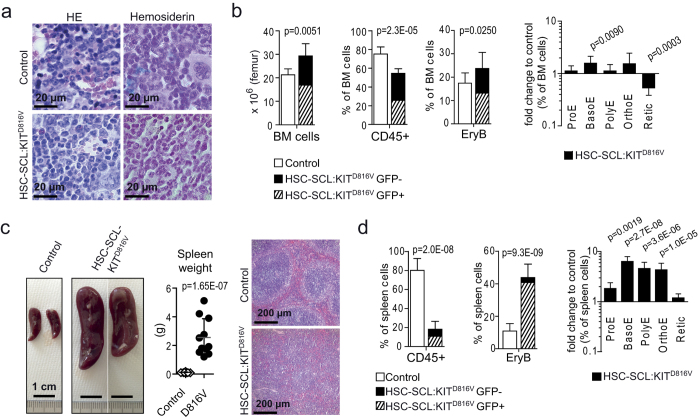
KIT^D816V^ induces excessive splenic stress erythropoiesis in HSC-SCL:KIT^D816V^ mice. Hematopoietic organs of HSC-SCL:KIT^D816V^ and control mice were analyzed 7–10 weeks after induction of KIT^D816V^ expression with TX. (**a**,**b**) Analysis of BM. (**a**) Paraffin sections were subjected to HE staining and Prussian Blue staining for hemosiderin. (**b**) Graphs show quantification of indicated cell populations. For HSC-SCL:KIT^D816V^ mice, the frequency of GFP-positive cells within each population is depicted. Right bar graph in Log10 scale. BM Cellularity - Control: N = 7. HSC-SCL:KIT^D816V^: N = 5. CD45-positive cells - Control: N = 9. HSC-SCL:KIT^D816V^: N = 7. Erythroid populations - Control: N = 11. HSC-SCL:KIT^D816V^: N = 8. (**c**,**d**) Analysis of the spleen. (**c**) Left: Macroscopic view. Scale bars: 1 cm. Middle: Scatter plot shows spleen weight in g. Control: N = 15. HSC-SCL:KIT^D816V^: N = 11. Right: HE stainings of paraffin sections. (**d**) Quantification of indicated cell populations in the spleen. Right graph in Log10 scale. CD45-positive cells - Control: N = 9. HSC-SCL:KIT^D816V^: N = 7. Erythroid populations - Control: N = 10. HSC-SCL:KIT^D816V^: N = 8. EryB: Erythroblasts; ProE: Proerythroblasts; BasoE: Basophilic EryB; PolyE: Polychromatic EryB; OrthoE: Orthochromatic EryB; Retic: Reticulocytes. Data are presented as mean ± standard deviation. P-values were determined using two-tailed, unpaired Student’s t-test.

BM and spleen were analyzed 10 weeks after KIT^D816V^ induction or when enlargement of the abdomen was observed. Hematoxylin and eosin (HE) staining revealed no apparent differences in BM histology of HSC-SCL:KIT^D816V^ and control animals. However, siderophages (mononuclear phagocytes containing hemosiderin, a product of hemoglobin catabolism) were scattered throughout control BM, whereas HSC-SCL:KIT^D816V^ mutants showed almost no siderophages, indicating reduced iron storage (Fig. 2a). Quantification further demonstrated a slight reduction in BM megakaryocytes. Immunohistochemical staining for active Caspase3 revealed a slight elevation of apoptosis in HSC-SCL:KIT^D816V^ BM, while Ki67 staining showed no differences in proliferation (Supplementary Fig. S3). Compared to controls, total BM cellularity of HSC-SCL:KIT^D816V^ mice was moderately increased (Fig. 2b). GFP-fluorescence was examined to estimate efficiency of KIT^D816V^ induction in HSC-SCL:KIT^D816V^ animals, demonstrating transgene expression in 57.40 ± 6.41% of total BM cells (Fig. 2b, dashed bar). While the frequency of CD45-positive cells was decreased in HSC-SCL:KIT^D816V^ BM, the overall frequency of CD45-negative erythroblasts was elevated in comparison to controls. Flow cytometric quantification of discrete developmental stages^19^,^27^ demonstrated a trend towards increased early and late erythroblasts, whereas reticulocytes were reduced, indicating a mild shift to more immature cells (Fig. 2b, Supplementary Fig. S3). To also investigate early BM erythropoiesis, we performed assays for CFU-E progenitors, showing no differences between HSC-SCL:KIT^D816V^ and control mice (Supplementary Fig. S3).

Gross examination of the mice revealed massive splenomegaly in HSC-SCL:KIT^D816V^ animals, another diagnostic criterion for PV^23^. KIT^D816V^ mutants showed a 19.81 ± 10.09-fold increase in spleen weight and altered spleen histology (Fig. 2c). Lymphoid nodules, normally marked by clusters of CD3-positive cells, were virtually absent in the spleen of mutant mice (Supplementary Fig. S3).

Flow cytometric examination demonstrated CD45 expression in almost 80% of control spleen cells. In HSC-SCL:KIT^D816V^ mice, this frequency was reduced to less than 20% (Fig. 2d). This reduction was due to a massive increase in splenic erythropoiesis, as CFU-Es and erythroblast frequency were significantly elevated in HSC-SCL:KIT^D816V^ mice (Figs 1c and 2d, Supplementary Fig. S3). The high ratio of GFP-positive erythroblasts substantiates that this expansion was KIT^D816V^-dependent.

In summary, these data demonstrate that chronic KIT^D816V^ signaling causes a PV-like disease based on moderately increased BM erythropoiesis and a massive induction of splenic erythropoiesis. As PV is often associated with high incidence of thrombosis due to elevated Hct^23^, we assume that abrupt death of HSC-SCL:KIT^D816V^ animals is a consequence of thrombotic events.

### KIT^D816V^ signaling causes stem cell mobilization from BM and a shift of hematopoiesis to extramedullary sites

**Figure 3:**
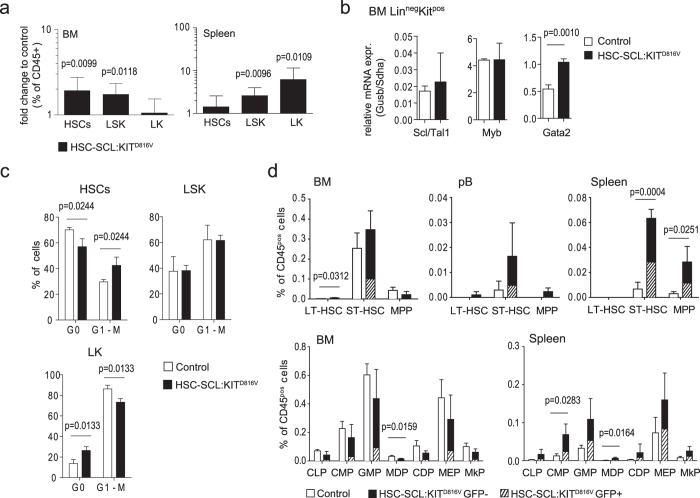
KIT^D816V^ mediates increased proliferation and *Gata2* expression and causes mobilization of BM HSCs and hematopoietic progenitor cells. Indicated populations bearing different stem cell potential were analyzed by flow cytometry 7–10 weeks after KIT^D816V^ induction in HSC-SCL:KIT^D816V^ and control mice. (**a**) Graphs illustrate the fold change in frequency of CD45-positive cells for HSC-SCL:KIT^D816V^ mice in relation to controls; Log10 scale. BM - Control: N = 11. HSC-SCL:KIT^D816V^: N = 8. Spleen - Control: N = 9. HSC-SCL:KIT^D816V^: N = 7. (**b**) The Lin^neg^Kit^pos^ cell population was purified from HSC-SCL:KIT^D816V^ and control BM 4 to 6 weeks after TX injection by flow cytometric cell sorting and analyzed for expression of indicated genes by quantitative real-time PCR. Data are presented as relative expression normalized to Gusb and Sdha. Control: N = 3. HSC-SCL:KIT^D816V^: N = 3. (**c**) Combined Ki67/DAPI staining was used to analyze the cell cycle status of indicated BM stem cell populations via flow cytometry. Control: N = 3. HSC-SCL:KIT^D816V^: N = 3. (**d**) The frequency of LT-HSCs, ST-HSCs, MPPs or defined progenitor cells within the CD45-positive cell population was analyzed for indicated hematopoietic compartments. Control: N = 3. HSC-SCL:KIT^D816V^: N = 3. CDP: Common dendritic progenitor. CLP: Common lymphoid progenitor. CMP: Common myeloid progenitor. GMP: Granulocyte-macrophage progenitor. HSC: Hematopoietic stem cell. LT-HSC: Long-term HSC. MDP: Macrophage-dendritic progenitor. MPP: Multipotent progenitor. MEP: Megakaryocyte-erythroid progenitor. MkP: Megakaryocyte progenitor. ST-HSC: Short-term HSC. Data are presented as mean ± standard deviation. P-values were determined using two-tailed, unpaired Student’s t-test.

We next examined effects of KIT^D816V^ signaling on stem cells by analyzing the frequencies of LK (KIT^pos^Sca-1^neg^Lin^neg^), LSK (KIT^pos^Sca-1^pos^Lin^neg^) and HSC (LSK-CD48^neg^CD150^pos^) populations among CD45-positive cells. BM HSC and LSK populations were increased in HSC-SCL:KIT^D816V^ animals compared to controls (Fig. 3a, Supplementary Fig. S4). To investigate the reasons for this expansion, we sorted Lin^neg^KIT^pos^ BM cells and analyzed expression of important transcriptional regulators. Increased *Gata2* transcript was found in the stem cell-enriched compartment of KIT^D816V^ mutants (Fig. 3b, Supplementary Fig. S4). Consistently, Gata2 has been shown to mediate proliferation and survival in hematopoietic stem cell compartments and *in vitro* differentiation of Gata2-deficient embryonic stem cells demonstrated impaired SCF-responsiveness^28^,^29^.

To examine if KIT^D816V^ signaling confers a proliferative advantage to stem cells, we analyzed Ki67 expression in BM stem cell populations (Fig. 3c, Supplementary Fig. S4). Interestingly, we found that the effects varied between the different populations. While actively cycling cells were increased in the HSC population of HSC-SCL:KIT^D816V^ animals, they were decreased in the LK population, suggesting that KIT stimulates proliferation in HSCs but supports cell cycle exit in the LK population. To see if we could find alterations in downstream signaling of KIT^D816V^ expressing stem cell populations, we stimulated BM cells with SCF or thrombopoietin and used phospho-flow cytometry to analyze phosphorylation of Erk1/2 and Akt in LK and LSK populations. However, for the analyzed pathways we could find no differences on the level of the examined cell populations (Supplementary Fig. S4). So, studies of additional pathways and more precisely defined cell populations will be necessary to find out more about signaling changes in KIT^D816V^ stem cells.

In the spleen, HSCs showed a mild but not significant elevation, while the LSK and LK populations bearing lower stem cell potential were significantly increased (Fig. 3a, Supplementary Fig. S4).

We further examined distribution of long-term (LT)-HSCs, short-term (ST)-HSCs and multipotent progenitors (MPPs) between BM, pB and spleen of HSC-SCL:KIT^D816V^ and control mice. We found BM LT-HSCs slightly increased in HSC-SCL:KIT^D816V^ mice, while the other populations in BM were not significantly altered. In contrast, ST-HSC and MPP frequencies were considerably elevated in pB and spleen (although changes were not significant for pB), indicating increased HSC activation and mobilization from BM to circulation. Consistently, progenitor cell populations were slightly reduced in BM but increased in spleen (Fig. 3d, Supplementary Fig. S4).

HSC analysis includes Kit as a cell surface marker. It is thus important to note that the KIT^D816V^ mutant receptor localizes to intracellular compartments^30^. In line with this, we found the fraction of cells positive for Kit surface expression similar in GFP-positive and GFP-negative BM populations in HSC-SCL:KIT^D816V^ mice, suggesting that only the endogenous receptor reaches the cell surface and ectopic KIT^D816V^ expression does not affect HSC analysis (Supplementary Fig. S5).

Unexpectedly, the frequency of GFP-positive cells was quite low in KIT^D816V^ HSCs, while it increased with progressive differentiation. To examine potential loss of KIT^D816V^-positive HSCs or high Cre-recombination frequencies in differentiated cells, we analyzed kinetics of reporter gene expression in different hematopoietic populations 2, 4 and 6 weeks after induction. The GFP-frequency was stable in the LT-HSC compartment and gradually increased in ST-HSC and MPP populations, indicating no negative influence of KIT^D816V^ signaling on stem cell survival (Supplementary Fig. S5). Initially, the GFP-frequency in most progenitor and mature populations was lower than in stem cells, demonstrating Cre-recombination primarily in HSCs, albeit at low frequency. However, analyses suggested also low recombination in granulocytes and B-cells. As the GFP-positive fraction was especially high in the erythroid compartment, we analyzed erythroblasts directly after TX-induction, further revealing recombination in proerythroblasts to a comparatively high degree (Supplementary Fig. S5).

**Figure 4:**
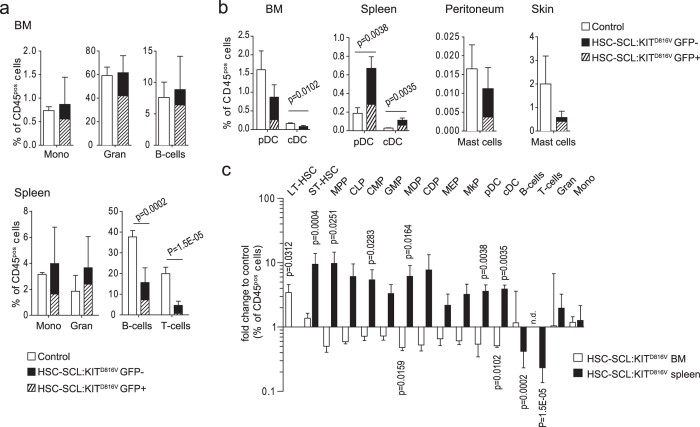
KIT^D816V^ induces a shift of hematopoiesis to the spleen. (**a**) BM and spleen cells from HSC-SCL:KIT^D816V^ and control mice were analyzed 7–9 weeks after TX-treatment for indicated populations via flow cytometry. BM - Control: N = 3. HSC-SCL:KIT^D816V^: N = 3. Spleen - Control: N = 5. HSC-SCL:KIT^D816V^: N = 5. (**b**) Dendritic cells and mast cell infiltrations were analyzed in indicated organs 7 weeks after TX-treatment via flow cytometry. Control: N = 3. HSC-SCL:KIT^D816V^: N = 3. DC: Dendritic cell. cDC: Conventional DC. pDC: Plasmacytoid DC. (**c**) Graph summarizes the alterations in analyzed HSC/HPC- and differentiated blood cell populations for BM and spleen of HSC-SCL:KIT^D816V^ mice. Data presented as fold change in frequency within the CD45-positive cell population for HSC-SCL:KIT^D816V^ mice in relation to controls; Log10 scale. CLP: Common lymphoid progenitor. CMP: Common myeloid progenitor. GMP: Granulocyte-macrophage progenitor. MDP: Macrophage-dendritic progenitor. CDP: Common dendritic progenitor. MEP: Megakaryocyte-erythroid progenitor. MkP: Megakaryocyte progenitor. Data are presented as mean ± standard deviation. P-values were determined using two-tailed, unpaired Student’s t-test.

As we found alterations in stem cell distribution, we also analyzed distribution of mature blood cells. In BM, we found no alterations in granulocyte, B-cell and monocyte populations, while in the spleen we found a reduction in lymphocyte frequency for HSC-SCL:KIT^D816V^ animals (Fig. 4a, Supplementary Fig. S6). Analysis of dendritic cells (DCs) revealed a decrease in BM and an increase in the spleen of HSC-SCL:KIT^D816V^ mice. As KIT^D816V^ is strongly associated with mastocytosis^8^,^9^, we checked for mast cell infiltrations. Surprisingly, flow cytometry revealed a reduced number of peritoneal and skin mast cells in mutants, although changes were not significant (Fig. 4b, Supplementary Fig. S6).

Taking together, our analyses demonstrate that stem cell and differentiated blood cell populations tend to be reduced in BM but increased in spleen (with the exception of lymphocytes), indicating a shift in hematopoiesis to extramedullary sites (Fig. 4c).

### The KIT^D816V^-mediated PV-like phenotype is transplantable

**Figure 5:**
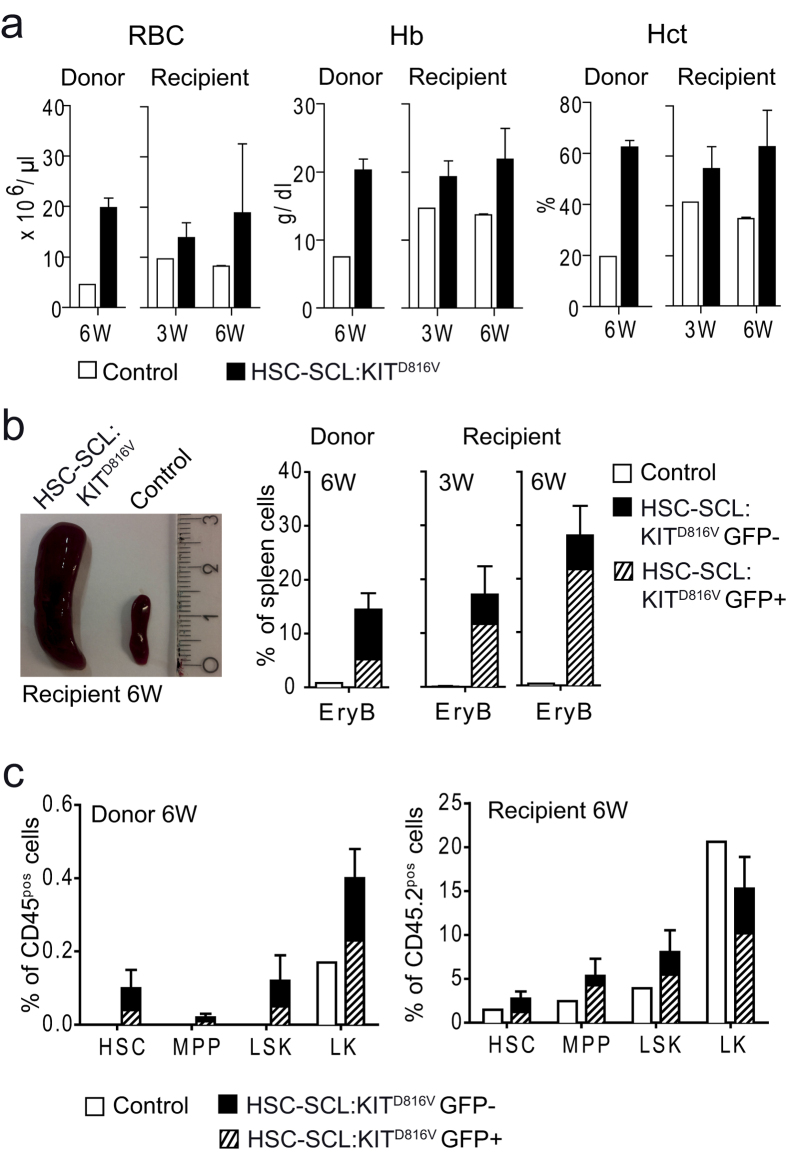
The KIT^D816V^-induced increase in red cell mass and extramedullary hematopoiesis is transplantable. Whole BM cells from HSC-SCL:KIT^D816V^ and control mice were used for BM transplantation 6 weeks after KIT^D816V^ induction. Recipients were analyzed 3 and 6 weeks post-transplantation. Control donors: N = 1. HSC-SCL:KIT^D816V^ donors: N = 3. 3 W - Control recipients: N = 1. HSC-SCL:KIT^D816V^ recipients: N = 3. 6 W - Control recipients: N = 2 for (**a**); N = 1 for (**b**,**c**). HSC-SCL:KIT^D816V^ recipients: N = 5. (**a**) Indicated pB parameters were analyzed with a hematology analyzer. (**b**) Representative macroscopic picture of the spleens of control and HSC-SCL:KIT^D816V^ recipients 6 weeks after transplantation. Graphs illustrate quantification of splenic erythroblasts (EryB) as analyzed by flow cytometry. (**c**) Quantification of splenic HSC/HPC populations shown as frequency of CD45-positive cells. For recipients of BM transplants, the frequency was calculated for the CD45.2-positive donor cell population. Data are presented as mean ± standard deviation.

In addition to the hematopoietic compartment, HSC-SCL-Cre-ER^T^-mediated recombination also occurs in endothelial cells^21^. Hence, KIT^D816V^ signaling might also be activated in cells of the perivascular niche. Thus, we investigated whether the PV-like phenotype was a secondary event due to changes in the hematopoietic microenvironment. We treated HSC-SCL:KIT^D816V^ and control animals with TX and performed transplantation of unfractionated BM 6 weeks later. Recipients were analyzed 3 and 6 weeks after transplantation. In HSC-SCL:KIT^D816V^ transplanted mice we observed a gradual increase in RBC, Hb, Hct and WBC (Fig. 5a, Supplementary Fig. S7). Moreover, HSC-SCL:KIT^D816V^ recipients developed splenomegaly and progressively elevated erythroblast numbers (Fig. 5b, Supplementary Fig. S7). While splenic stem cell frequencies were generally elevated post-transplantation, absolute numbers were markedly higher for HSC-SCL:KIT^D816V^ recipients compared to controls (Fig. 5c, Supplementary Fig. S7). In summary, these data demonstrate that early PV-like disease develops autonomously from the medullary hematopoietic compartment.

### Splenectomy protects HSC-SCL:KIT^D816V^ mice from increased red blood cell mass but promotes rapid hematopoietic failure

**Figure 6:**
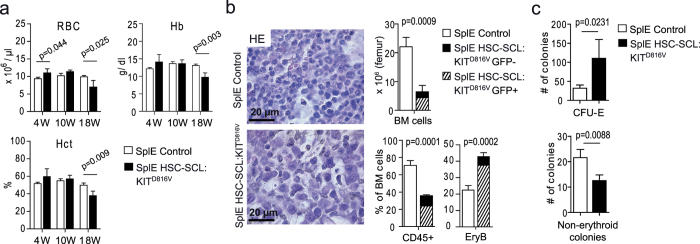
Splenectomy rescues HSC-SCL:KIT^D816V^ mice from excessive production of mature blood cells but promotes development of anemia and BM cell loss. HSC-SCL:KIT^D816V^ and control mice were subjected to SplE, followed by TX-treatment. (**a**) Indicated pB parameters were analyzed 4, 10 and 18 weeks after KIT^D816V^ induction with a hematology analyzer. SplE Control: N = 4. SplE HSC-SCL:KIT^D816V^: N = 3. (**b**,**c**) Analyses performed 18 weeks after KIT^D816V^ induction. SplE Control: N = 4. SplE HSC-SCL:KIT^D816V^: N = 3. (**b**) Pictures show BM paraffin sections that were subjected to HE staining. The upper graph depicts quantification of the absolute cell number in BM. For the lower graphs, nucleated pB cells were analyzed via flow cytometry for CD45 and erythroid marker proteins. EryB: Erythroblasts. For HSC-SCL:KIT^D816V^ mice, the frequency of GFP-positive cells within each population is indicated. (**c**) CFC assays performed with BM cells. Data are presented as mean ± standard deviation. P-values were determined using two-tailed, unpaired Student’s t-test.

We assumed that the high mortality rate of HSC-SCL:KIT^D816V^ animals was a consequence of thrombotic events caused by the elevated Hct due to massive splenic erythropoiesis. We therefore hypothesized that splenectomy (SplE) might have a protective effect and subjected HSC-SCL:KIT^D816V^ and control animals to SplE prior to TX-treatment (afterwards termed SplE HSC-SCL:KIT^D816V^ and SplE control mice). Blood parameters determined 4 and 10 weeks after KIT^D816V^ induction demonstrated that SplE HSC-SCL:KIT^D816V^ mice indeed were protected from excessive red blood cell production, as RBC, Hb, Hct, MCV and reticulocyte frequency were similar to SplE controls (Fig. 6a, Supplementary Fig. S8). Moreover, although pB monocytes were significantly elevated after 18 weeks, the WBC and the overall number of CD45-positive cells were unaffected in SplE HSC-SCL:KIT^D816V^ animals (Supplementary Fig. S8). Surprisingly, however, 18 weeks after induction SplE HSC-SCL:KIT^D816V^ animals became anemic, with RBC, Hb, Hct and PLT values falling significantly below control values (Fig. 6a, Supplementary Fig. S8). Despite the decrease in erythrocytes, a considerable number of erythroblasts was found in pB of SplE HSC-SCL:KIT^D816V^ mice (Supplementary Fig. S8).

Histologically, BM of SplE HSC-SCL:KIT^D816V^ mice appeared hypocellular compared to SplE controls. Consistently, the total cell number per femur and BM megakaryocyte counts were dramatically decreased (Fig. 6b, Supplementary Fig. S8). In SplE controls BM siderophages were abnormally abundant, indicating partial take-over of red blood cell destruction after spleen removal. In contrast, siderophages were absent in KIT^D816V^ mutants after SplE, again demonstrating affected iron storage (Supplementary Fig. S8).

After 18 weeks we found the frequency of CD45-positive cells in SplE HSC-SCL:KIT^D816V^ BM considerably reduced in favor of a significant increase in erythroblast frequency (Fig. 6b). However, an elevated ratio of early and late erythroblasts to reticulocytes indicated ineffective cell maturation. Moreover, given the overall cell loss in BM, total cell numbers were reduced for all erythroid populations (Supplementary Fig. S8). In line with the increase in erythroblast frequency, CFC-assays revealed significantly more CFU-E colonies for SplE HSC-SCL:KIT^D816V^ animals compared to SplE controls. In contrast, the number of non-erythroid colonies was reduced for SplE HSC-SCL:KIT^D816V^ mice (Fig. 6c). These data indicate, that KIT^D816V^ preferentially promotes erythropoiesis.

As extramedullary hematopoiesis can also occur in liver, SplE animals were examined for liver erythropoiesis (Supplementary Fig. S8). Indeed, a mild induction of erythropoiesis was found in livers of SplE HSC-SCL:KIT^D816V^ mice, but the overall extent was low and no hepatomegaly was observed.

### Splenectomy is accompanied by stem cell loss and BM fibrosis in KIT^D816V^ mice

**Figure 7:**
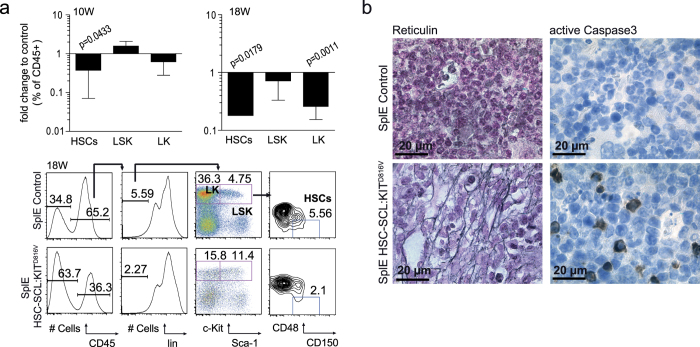
KIT^D816V^ induces fibrosis and loss of stem cell populations in BM of SplE HSC-SCL:KIT^D816V^ mice. HSC-SCL:KIT^D816V^ and control mice were subjected to SplE, followed by TX-treatment. (**a**) Indicated BM cell populations were analyzed 10 and 18 weeks after induction. Graphs show fold change in frequency of CD45-positive cells for SplE HSC-SCL:KIT^D816V^ mice in relation to controls; Log10 scale. Representative flow cytometric panels from 18 weeks analysis are depicted. 10 weeks - SplE Control: N = 4. SplE HSC-SCL:KIT^D816V^: N = 3. 18 weeks - SplE Control: N = 4. SplE HSC-SCL:KIT^D816V^: N = 3. (**b**) 18 weeks after induction BM paraffin sections were analyzed by reticulin staining and immunohistological staining against active Caspase3. Reticulin staining demonstrated BM fibrosis in all analyzed SplE HSC-SCL:KIT^D816V^ mice (10 weeks N = 3/18 weeks N = 3). In 18 weeks samples a loose network of reticulin fibers was found (MF-1–MF-2 according to the European Consensus fibrosis grading). Data are presented as mean ± standard deviation. P-values were determined using two-tailed, unpaired Student’s t-test.

Analyzing stem cell populations of SplE HSC-SCL:KIT^D816V^ animals, we found a strong progressive reduction in BM HSCs/HPCs compared to SplE controls (Fig. 7a, Supplementary Fig. S9). This was in contrast to KIT^D816V^ mutants not subjected to SplE, which showed no indication of stem cell depletion after 10 weeks (Fig. 3a). We also examined Erk1/2 and Akt phosphorylation in BM LK and LSK populations of SplE animals, but again found no constitutive activation or altered reaction to SCF or thrombopoietin stimulation in KIT^D816V^ cells (Supplementary Fig. S9).

Our results demonstrate, that although SplE protects HSC-SCL:KIT^D816V^ mice from excessive red blood cell accumulation, it promotes rapid BM failure. Clinically, PV can progress to its spent phase polycythemic myeloid metaplasia (PPMM)^31^. There, the initially hypercellular BM becomes hypocellular and secondary myelofibrosis develops. Red blood cell production becomes ineffective and pB cell counts decrease, resembling aplastic anemia. In this study, HSC-SCL:KIT^D816V^ mice without SplE developed a malignancy reminiscent of early PV, whereas the phenotype observed in SplE HSC-SCL:KIT^D816V^ mice resembled PPMM. To further validate this, we performed staining for reticulin fibers to check for BM fibrosis. Indeed, fibrotic changes were found in SplE HSC-SCL:KIT^D816V^ BM 10 and 18 weeks after KIT^D816V^ induction^32^. In contrast, HSC-SCL:KIT^D816V^ mice not subjected to SplE neither displayed any signs of fibrosis in BM after 10 or 18 weeks nor increased reticulin deposition in spleen (Fig. 7b, Supplementary Fig. S9). In SplE HSC-SCL:KIT^D816V^ BM we also observed considerably elevated apoptosis, analyzed by immunostaining for active Caspase3 (Fig. 7b). Ki67 staining showed no apparent differences (Supplementary Fig. S9).

Our results indicate that removal of the splenic hematopoietic niche in KIT^D816V^ mutants dramatically influences the clinical picture of the PV-like disease.

## Discussion

In this study, we analyzed the consequences of oncogenic KIT^D816V^ expression in the adult hematopoietic system and found MPN development reminiscent of early and advanced forms of PV, depending on pre-treatment of animals with SplE.

Upon induction of KIT^D816V^ expression, we observed massively increased red blood cell production. Under normal conditions, the erythrocyte pool is maintained by BM steady-state erythropoiesis, whereas extramedullary stress erythropoiesis mediates its rapid expansion upon acute anemia^33^. Different studies have reported a role of KIT in stress erythropoiesis^34^,^35^. Accordingly, we found the increase in red blood cells to depend on splenic erythropoiesis. Similar observations have been reported for KIT^V558∆;T669I/+^ mice^36^. While this clearly demonstrates involvement of KIT in stress erythropoiesis, effects on BM steady-state erythropoiesis are difficult to evaluate in our model, since analysis is impeded by splenic erythropoiesis or hematopoietic failure. However, the erythroid expansion in BM of SplE KIT^D816V^ mutants indicates that KIT signaling regulates proliferative expansion during both, steady-state and stress erythropoiesis.

HSC-SCL:KIT^D816V^ mice had significantly increased pB monocytes, which is likely based on increased proliferation rather than mobilization, as the monocyte frequency in HSC-SCL:KIT^D816V^ BM or spleen^37^ was not reduced and pB monocytes were also elevated after SplE. Detailed investigation will be necessary to elucidate effects of KIT^D816V^ on the monocytic lineage. Untypical for PV, pB B-cells were also elevated in HSC-SCL:KIT^D816V^ mice. We suppose that this was primarily caused by displacement from the spleen, which showed markedly reduced B-cell frequency.

In HSC-SCL:KIT^D816V^ animals we observed mobilization of HSCs to the spleen. Several studies have indicated a role of KIT in mobilization of stem cells from the quiescent niche^38^,^39^. Mobilization of HSCs/HPCs from the BM niche was shown to depend on the release of soluble SCF mediated by matrix metalloproteinase-9 (MMP9)^38^. Moreover, KIT signaling is involved in cell mobilization triggered by functionally blocking cytoadhesion molecules VLA4/VCAM-1, suggesting an integrin/cytokine crosstalk^39^. We also observed an increased frequency of cycling HSCs and increased ST-HSCs in KIT^D816V^ mutants. Differences in the KIT expression level within the LSK-CD150^pos^CD48^neg^ HSC pool have been reported, with lower expression in quiescent cells^40^. These data indicate that oncogenic KIT^D816V^ contributes to activation and mobilization of dormant HSCs. Interestingly, mobilization of HSCs/HPCs has also been associated with primary myelofibrosis and PPMM^41^,^42^,^43^.

HSC-SCL:KIT^D816V^ animals pre-treated by SplE showed stem cell loss and myelofibrosis already 10 weeks after KIT^D816V^ induction. Interestingly, no fibrosis was detected in HSC-SCL:KIT^D816V^ mice without SplE, suggesting that disease pathogenesis depends not only on time but also on the interplay between KIT^D816V^-positive cells and the medullary and extramedullary hematopoietic niches. This is in line with the “bad seeds in bad soil” concept proposed for primary myelofibrosis, presuming that an abnormal hematopoietic cell clone alters its microenvironment, resulting in niche dysfunctions^42^. One may hypothesize, that abnormal KIT^D816V^-positive cells cycle between hematopoietic niches and upon splenectomy remain in or repopulate the BM and produce high levels of fibrogenic cytokines, stimulating stromal reticulin production. Consequently, displacement from the niche caused by KIT^D816V^-induced stem cell mobilization and myelofibrosis leads to hematopoietic failure. Studies which have shown that the spleen serves as a reservoir of aberrant stem cells in primary myelofibrosis patients support this assumption^44^,^45^. Further, a study by Migliaccio *et al*. demonstrated that the spleen microenvironment is capable of supporting maturation of Gata1^low^ mutant stem cells that fail to mature in the BM, suggesting the possibility that the BM niche may likewise not sustain maturation of KIT^D816V^ mutant cells^46^.

Future experiments with SplE HSC-SCL:KIT^D816V^ mice should investigate the disease-promoting cell population(s) and cytokine production to further substantiate this hypothesis.

As SplE can manipulate the course of disease, HSC-SCL:KIT^D816V^ mice provide an excellent model to study the interplay between hematopoietic cells and microenvironment in PV-like disease.

Noteworthy, while Philadelphia-negative MPN are highly associated with the JAK2^V617F^ mutation found in hematopoietic and endothelial-like cells^47^,^48^, there is only one study reporting an association of KIT mutations with PV^49^. However, other sequencing studies did not confirm this association, raising the question for the relevance of activating KIT mutations in human MPN. It might be possible that alternative mechanisms lead to altered KIT signaling in human MPN. For instance, it was shown that cultured pB cells from primary myelofibrosis patients produce elevated levels of activated MMP9^50^. As mentioned before, MMP9 can mediate release of soluble KIT ligand^38^. Furthermore, phospho-proteomic analysis of erythroblasts from PV patients revealed reduced total KIT and phospho-KIT-Y719 protein content, indicating altered KIT signaling^51^. It remains unclear if this results from hyper- or hypoactivation of the pathway, as the relative KIT-Y719 phosphorylation in PV and control groups was not compared. However, the reduction in total KIT on protein but not transcript level points to increased internalization upon activation in PV cells.

KIT^D816V^ represents a frequent mutation in mastocytosis in man^8^,^9^ and development of cutaneous mastocytosis with variable speed and degree has been demonstrated in a BAC transgenic Kit^D814V^ mouse model^52^. Thus, it was surprising that peritoneal and skin mast cells were reduced in HSC-SCL:KIT^D816V^ mice and no mastocytosis was observed. This discrepancy might be due to differential expression levels, as the the *Kit* promoter exhibits strong physiological activity in the mast cell lineage^15^,^53^ compared to moderate *ROSA26-*mediated expression. Moreover, mastocytosis in man is associated with additional mutations, such as Tet2, Srsf2, Asxl1, Cbl and Runx1, which are not present in HSC-SCL:KIT^D816V^ mice^54^,^55^. A recent study by Jawhar *et al*. has shown that mutations in TET2, SRSF2 or ASXL1 precede the KIT^D816V^ mutation in mastocytosis^56^. Thus, in the absence of such seed mutations the KIT^D816V^ mediated disease phenotype might well be different from the phenotype in KIT^D816V^ mastocytosis patients. In addition to mastocytosis, the KIT^D816V^ mutation is also frequently identified in the core binding factor leukemias, involving the AML1-ETO or CBFB/MYH11 genes (t[8;21] or inv[16]/t[16;16], respectively)^7^,^57^. A study by Wang *et al*. found strong evidence that in t(8;21) AML, AML1-ETO is the first genetic hit which is responsible for disease initiation, while KIT^D816V^ is a secondary mutation^58^.

Features reminiscent of early PV have also been observed in KIT^V558∆;T669I/+^ mice^36^,^59^. However, the effects were rather mild, probably due the endogenous *Kit* promoter driving the mutated KIT, mediating no expression at later developmental stages. We show that KIT^D816V^ expression driven by the *ROSA26* promoter causes a more severe and complex phenotype (PPMM/myelofibrosis). We speculate that our KIT^D816V^ allele, which is not downregulated upon differentiation might better mirror pathology of some MPN entities, where deregulated gene expression patterns are found^60^,^61^,^62^. Taking together, R26-LSL-KIT^D816V^ mice provide a valuable model to investigate interactions of aberrant KIT^D816V^ cells with the hematopoietic microenvironment leading to PV-like disease and myelofibrosis.

## Materials and Methods

### Animal studies

All experiments were compliant with the German law of animal protection and the European Directive 86/609/EEC and were approved by the local institutional animal care committees (Landesamt für Natur, Umwelt und Verbraucherschutz, North Rhine-Westphalia; approval-ID: #84-02.04.2013.A491/#84-02.04.2012.A256).

R26-LSL-KIT^D816V^ mice^19^ were maintained on 129 Sv/S2, HSC-SCL-Cre-ER^T^ mice^21^ on C57BL/6 genetic background. For transgene induction animals received intraperitoneal injection of 1.5 mg TX/day for 5 consecutive days at a minimum age of 8 weeks. Peripheral blood samples were collected from the tail vein. Blood and BM cell counts were determined using an Hemavet 950 hematology analyzer (Drew Scientific, Miami Lakes, FL, USA). Studies were powered to detect a pre-specified RBC difference of 1 × 10^6^ cells/μl between HSC-SCL:KIT^D816V^ and control animals (effect size = 1.48, two-sided *α*-value = 0.05/ß-value = 0.2). For splenectomy, animals were anesthetized and an incision was made at the left abdominal wall. Ligatures were set around splenic vessels before spleen removal. The incision was closed with wound clips. Following surgery, animals obtained carprofen for pain relief and were allowed to recover for one week before TX-treatment. For transplantation assays, R26-LSL-KIT^D816V^ mice were backcrossed to C57BL/6. CD45.1 recipient mice were subjected to lethal irradiation (2 × 5.5 Gray in 4 h interval) and 2 × 10^6^ unfractionated BM cells from HSC-SCL:KIT^D816V^ CD45.2 donor mice were transplanted via tail vein injection. Reconstitution of CD45.2 donor cells was analyzed by flow cytometry. Splenectomy/transplantation studies were powered to detect a RBC difference of 7 × 10^6^ cells/μl, based on earlier results (effect size = 4.69).

### Colony forming cell assays and erythropoietin levels

Colony forming cell (CFC) assays were performed in technical triplicates using MethoCult methylcellulose medium (StemCell Technologies, Vancouver, Canada) according to manufacturers instructions with minor modifications. Per replicate, 5 × 10^4^ nucleated cells were plated for BM and 2.5 × 10^5^ for spleen. CFU-E colonies were scored after 2–4 days. If not stated otherwise, erythropoietin concentration was 3 U/ml. For GEMM and GM colonies, the medium was supplemented with 50 ng/ml SCF, 10 ng/ml IL3 and 10 ng/ml IL6 and the total colony number scored after 9–10 days. Serum erythropoietin and thrombopoietin levels were determined by ELISA (R&D Systems, Minneapolis, MN, USA).

### Flow cytometry

For flow cytometry, cells were stained with fluorochrome-conjugated antibodies listed in Supplementary Table S1 or with 1 μg/ml Hoechst H33342 for staining of nucleated cells. For reticulocyte staining, whole EDTA blood was stained using anti-Ter119-antibody and 1 ng/ml thiazol orange. Flow cytometry was performed on a BD FACSCanto™ or a BD FACSCanto™ II Flow Cytometer (Becton Dickinson, Heidelberg, Germany). Data were analyzed using FlowJo software (TreeStar, Ashland, OR, USA). For marker combinations defining cell populations see Figure descriptions and Supplementary Table S2.

### Quantitative real-time PCR (qRT-PCR)

Total RNA was isolated using RNeasy Mini or RNeasy Micro Kits (Qiagen, Hilden, Germany). cDNA synthesis was performed using RevertAid Premium reverse transcriptase (Fermentas, Thermo Fisher Scientific, Waltham, MA, USA). For amplification of target sequences Maxima SYBR Green/ROX (Fermentas) was used. Reference genes *Gusb* (glucuronidase) and *Sdha* (succinate dehydrogenase) were used for normalization. Primer sequences for qRT-PCR are given in Supplementary Table S3.

### Histology and Immunohistochemistry

For histology, samples were fixed in 4% paraformaldehyde, dehydrated and paraffin-embedded. Immunohistochemistry was performed using the BrightVision Ultimate Plus Kit (Immunologic, Duiven, Netherlands) with antibodies listed in Supplementary Table 1. For Prussian Blue/hemosiderin staining, sections were deparaffinized, hydrated, stained in 1:2 potassium ferrocyanide (1–2%)/hydrochloric acid (1–2%) and counterstained with nuclear fast red. Reticulin fibers were stained using Gordon and Sweet’s silver staining protocol for reticulin^63^.

### Statistical analysis

All data are presented as mean ± standard deviation. P-values were determined using two-tailed, unpaired Student’s t-test. Studies were neither randomized nor blinded, as all animal experiments were performed with homogeneous age, strain and similar variance.

## Additional Information

**How to cite this article:** Pelusi, N. *et al*. The spleen microenvironment influences disease transformation in a mouse model of KIT^D816V^-dependent myeloproliferative neoplasm. *Sci. Rep.* **7**, 41427; doi: 10.1038/srep41427 (2017).

**Publisher's note:** Springer Nature remains neutral with regard to jurisdictional claims in published maps and institutional affiliations.

## Supplementary Material

Supplementary Information

## References

[b1] Ikuta K. & Weissman I. L. Evidence that hematopoietic stem cells express mouse c-kit but do not depend on steel factor for their generation. Proc. Natl. Acad. Sci. USA 89, 1502–6 (1992). 1371359 10.1073/pnas.89.4.1502 PMC48479

[b2] Ogawa M. . Expression and function of c-kit in hemopoietic progenitor cells. J. Exp. Med. 174, 63–71 (1991). 1711568 10.1084/jem.174.1.63 PMC2118893

[b3] Munugalavadla V. . Repression of c-kit and its downstream substrates by GATA-1 inhibits cell proliferation during erythroid maturation. Mol Cell Biol 25, 6747–6759 (2005). 16024808 10.1128/MCB.25.15.6747-6759.2005 PMC1190349

[b4] Lyman S. D. & Jacobsen S. E. W. c-kit ligand and Flt3 ligand: stem/progenitor cell factors with overlapping yet distinct activities. Blood 91, 1101–34 (1998). 9454740

[b5] Thorén L. a. . Kit regulates maintenance of quiescent hematopoietic stem cells. J. Immunol. 180, 2045–53 (2008). 18250409 10.4049/jimmunol.180.4.2045

[b6] Waskow C., Paul S., Haller C., Gassmann M. & Rodewald H.-R. Viable c-Kit(W/W) mutants reveal pivotal role for c-kit in the maintenance of lymphopoiesis. Immunity 17, 277–88 (2002). 12354381 10.1016/s1074-7613(02)00386-2

[b7] Kim H.-J. . KIT D816 mutation associates with adverse outcomes in core binding factor acute myeloid leukemia, especially in the subgroup with RUNX1/RUNX1T1 rearrangement. Ann. Hematol. 92, 163–71 (2013). 23053179 10.1007/s00277-012-1580-5

[b8] Bodemer C. . Pediatric mastocytosis is a clonal disease associated with D816V and other activating c-KIT mutations. J. Invest. Dermatol. 130, 804–15 (2010). 19865100 10.1038/jid.2009.281

[b9] Longley B. J. . Activating and dominant inactivating c-KIT catalytic domain mutations in distinct clinical forms of human mastocytosis. Proc. Natl. Acad. Sci. USA 96, 1609–14 (1999). 9990072 10.1073/pnas.96.4.1609 PMC15534

[b10] Anderson D. M. . Molecular cloning of mast cell growth factor, a hematopoietin that is active in both membrane bound and soluble forms. Cell 63, 235–43 (1990). 1698558 10.1016/0092-8674(90)90304-w

[b11] Zsebo K. M. . Stem cell factor is encoded at the Sl locus of the mouse and is the ligand for the c-kit tyrosine kinase receptor. Cell 63, 213–24 (1990). 1698556 10.1016/0092-8674(90)90302-u

[b12] Copeland N. G. . Mast cell growth factor maps near the steel locus on mouse chromosome 10 and is deleted in a number of steel alleles. Cell 63, 175–83 (1990). 1698554 10.1016/0092-8674(90)90298-s

[b13] Kimura Y. . c-Kit-mediated functional positioning of stem cells to their niches is essential for maintenance and regeneration of adult hematopoiesis. PLoS One 6, e26918 (2011). 22046410 10.1371/journal.pone.0026918 PMC3202594

[b14] Driessen R. L., Johnston H. M. & Nilsson S. K. Membrane-bound stem cell factor is a key regulator in the initial lodgment of stem cells within the endosteal marrow region. Exp. Hematol. 31, 1284–91 (2003). 14662336 10.1016/j.exphem.2003.08.015

[b15] Maeda K., Nishiyama C., Ogawa H. & Okumura K. GATA2 and Sp1 positively regulate the c-kit promoter in mast cells. J. Immunol. 185, 4252–60 (2010). 20833840 10.4049/jimmunol.1001228

[b16] de Aberle S. B. A study of the hereditary anaemia of mice. Am. J. Anat. 40, 219–249 (1927).

[b17] Muta K., Krantz S. B., Bondurant M. C. & Dai C. H. Stem cell factor retards differentiation of normal human erythroid progenitor cells while stimulating proliferation. Blood 86, 572–80 (1995). 7541668

[b18] Malaise M., Steinbach D. & Corbacioglu S. Clinical implications of c-Kit mutations in acute myelogenous leukemia. Curr Hematol Malig Rep 4, 77–82 (2009). 20425418 10.1007/s11899-009-0011-8

[b19] Haas N. . Kit transduced signals counteract erythroid maturation by MAPK-dependent modulation of erythropoietin signaling and apoptosis induction in mouse fetal liver. Cell Death Differ. 22, 790–800 (2015). 25323585 10.1038/cdd.2014.172 PMC4392076

[b20] Sakuma Y., Sakurai S., Oguni S., Hironaka M. & Saito K. Alterations of the c-kit gene in testicular germ cell tumors. Cancer Sci. 94, 486–91 (2003). 12824871 10.1111/j.1349-7006.2003.tb01470.x PMC11160296

[b21] Göthert J. R. . *In vivo* fate-tracing studies using the Scl stem cell enhancer: embryonic hematopoietic stem cells significantly contribute to adult hematopoiesis. Blood 105, 2724–32 (2005). 15598809 10.1182/blood-2004-08-3037

[b22] Zhang J., Socolovsky M., Gross A. W. & Lodish H. F. Role of Ras signaling in erythroid differentiation of mouse fetal liver cells: functional analysis by a flow cytometry-based novel culture system. Blood 102, 3938–46 (2003). 12907435 10.1182/blood-2003-05-1479

[b23] Spivak J. L. Polycythemia vera: myths, mechanisms, and management. Blood 100, 4272–90 (2002). 12393615 10.1182/blood-2001-12-0349

[b24] Wernig G. . EXEL-8232, a small-molecule JAK2 inhibitor, effectively treats thrombocytosis and extramedullary hematopoiesis in a murine model of myeloproliferative neoplasm induced by MPLW515L. Leukemia 26, 720–7 (2012). 22005786 10.1038/leu.2011.261

[b25] Wernig G. . Expression of Jak2V617F causes a polycythemia vera-like disease with associated myelofibrosis in a murine bone marrow transplant model. Blood 107, 4274–81 (2006). 16478879 10.1182/blood-2005-12-4824 PMC1895786

[b26] Zaleskas V. M. . Molecular pathogenesis and therapy of polycythemia induced in mice by JAK2 V617F. PLoS One 1, e18 (2006). 17183644 10.1371/journal.pone.0000018 PMC1762384

[b27] Sprüssel A. . Lysine-specific demethylase 1 restricts hematopoietic progenitor proliferation and is essential for terminal differentiation. Leukemia 26, 2039–51 (2012). 22699452 10.1038/leu.2012.157

[b28] Rodrigues N. P. . Haploinsufficiency of GATA-2 perturbs adult hematopoietic stem-cell homeostasis. Blood 106, 477–485 (2005). 15811962 10.1182/blood-2004-08-2989

[b29] Tsai F. Y. . An early haematopoietic defect in mice lacking the transcription factor GATA-2. Nature 371, 221–6 (1994). 8078582 10.1038/371221a0

[b30] Xiang Z., Kreisel F., Cain J., Colson A. & Tomasson M. H. Neoplasia driven by mutant c-KIT is mediated by intracellular, not plasma membrane, receptor signaling. Mol Cell Biol 27, 267–282 (2007). 17060458 10.1128/MCB.01153-06 PMC1800644

[b31] Mesa R. A., Elliott M. A. & Tefferi A. Splenectomy in chronic myeloid leukemia and myelofibrosis with myeloid metaplasia. Blood Rev. 14, 121–9 (2000). 10986148 10.1054/blre.2000.0132

[b32] Gianelli U. . The European Consensus on grading of bone marrow fibrosis allows a better prognostication of patients with primary myelofibrosis. Mod. Pathol. 25, 1193–1202 (2012). 22627739 10.1038/modpathol.2012.87

[b33] Paulson R. F., Shi L. & Wu D.-C. Stress erythropoiesis: new signals and new stress progenitor cells. Curr. Opin. Hematol. 18, 139–45 (2011). 21372709 10.1097/MOH.0b013e32834521c8 PMC3099455

[b34] Agosti V., Karur V., Sathyanarayana P., Besmer P. & Wojchowski D. M. A KIT juxtamembrane PY567 -directed pathway provides nonredundant signals for erythroid progenitor cell development and stress erythropoiesis. Exp. Hematol. 37, 159–171 (2009). 19100679 10.1016/j.exphem.2008.10.009 PMC2701661

[b35] Perry J. M., Harandi O. F. & Paulson R. F. BMP4, SCF, and hypoxia cooperatively regulate the expansion of murine stress erythroid progenitors. Blood 109, 4494–502 (2007). 17284534 10.1182/blood-2006-04-016154 PMC1885504

[b36] Deshpande S. . Kit receptor gain-of-function in hematopoiesis enhances stem cell self-renewal and promotes progenitor cell expansion. Stem Cells 31, 1683–1695 (2013). 23681919 10.1002/stem.1419 PMC3775897

[b37] Swirski F. K. . Identification of splenic reservoir monocytes and their deployment to inflammatory sites. Science 325, 612–6 (2009). 19644120 10.1126/science.1175202 PMC2803111

[b38] Heissig B. . Recruitment of stem and progenitor cells from the bone marrow niche requires MMP-9 mediated release of kit-ligand. Cell 109, 625–37 (2002). 12062105 10.1016/s0092-8674(02)00754-7 PMC2826110

[b39] Papayannopoulou T., Priestley G. V. & Nakamoto B. Anti-VLA4/VCAM-1-induced mobilization requires cooperative signaling through the kit/mkit ligand pathway. Blood 91, 2231–9 (1998). 9516120

[b40] Matsuoka Y. . Low level of c-kit expression marks deeply quiescent murine hematopoietic stem cells. Stem Cells 29, 1783–91 (2011). 21898688 10.1002/stem.721

[b41] Passamonti F. . Relation between JAK2 (V617F) mutation status, granulocyte activation, and constitutive mobilization of CD34+cells into peripheral blood in myeloproliferative disorders. Blood 107, 3676–82 (2006). 16373657 10.1182/blood-2005-09-3826

[b42] Le Bousse-Kerdilès M.-C. Primary myelofibrosis and the ‘bad seeds in bad soil’ concept. Fibrogenesis Tissue Repair 5, S20 (2012). 23259918 10.1186/1755-1536-5-S1-S20 PMC3368798

[b43] Lataillade J.-J. . Does primary myelofibrosis involve a defective stem cell niche? From concept to evidence. Blood 112, 3026–35 (2008). 18669872 10.1182/blood-2008-06-158386

[b44] Wang X. . Spleens of myelofibrosis patients contain malignant hematopoietic stem cells. J. Clin. Invest. 122, 3888–99 (2012). 23023702 10.1172/JCI64397 PMC3484080

[b45] O’Malley D. P., Orazi A., Wang M. & Cheng L. Analysis of loss of heterozygosity and X chromosome inactivation in spleens with myeloproliferative disorders and acute myeloid leukemia. Mod. Pathol. 18, 1562–8 (2005). 16118625 10.1038/modpathol.3800481

[b46] Migliaccio A. R. . Gata1 expression driven by the alternative HS2 enhancer in the spleen rescues the hematopoietic failure induced by the hypomorphic Gata1 low mutation. Blood 114, 2107–2121 (2009). 19571316 10.1182/blood-2009-03-211680 PMC2744572

[b47] Levine R. L. . Activating mutation in the tyrosine kinase JAK2 in polycythemia vera, essential thrombocythemia, and myeloid metaplasia with myelofibrosis. Cancer Cell 7, 387–97 (2005). 15837627 10.1016/j.ccr.2005.03.023

[b48] Sozer S. . Human CD34+cells are capable of generating normal and JAK2V617F positive endothelial like cells *in vivo*. Blood Cells. Mol. Dis. 43, 304–12 (2009). 19762257 10.1016/j.bcmd.2009.08.005

[b49] Fontalba A. . Identification of c-Kit gene mutations in patients with polycythemia vera. Leuk. Res. 30, 1325–6 (2006). 16460801 10.1016/j.leukres.2005.12.020

[b50] Ciurea S. O. . Pivotal contributions of megakaryocytes to the biology of idiopathic myelofibrosis. Blood 110, 986–993 (2007). 17473062 10.1182/blood-2006-12-064626 PMC1924766

[b51] Hricik T. . Transcriptomic and phospho-proteomic analyzes of erythroblasts expanded *in vitro* from normal donors and from patients with polycythemia vera. Am. J. Hematol. 88, 723–729 (2013). 23720412 10.1002/ajh.23487 PMC3771389

[b52] Gerbaulet A. . Mast cell hyperplasia, B-cell malignancy, and intestinal inflammation in mice with conditional expression of a constitutively active kit. Blood 117, 2012–21 (2011). 21148330 10.1182/blood-2008-11-189605

[b53] Orfao A. . Flow Cytometric Analysis of Mast Cells from Normal and Pathological Human Bone Marrow Samples. Am. J. Pathol. 149, 1493–1499 (1996). 8909239 PMC1865253

[b54] Schwaab J. . Comprehensive mutational profiling in advanced systemic mastocytosis. Blood 122, 2460–2466 (2013). 23958953 10.1182/blood-2013-04-496448

[b55] Jawhar M. . Additional mutations in SRSF2, ASXL1 and/or RUNX1 identify a high-risk group of patients with KIT D816V(+) advanced systemic mastocytosis. Leukemia 30, 136–43 (2016). 26464169 10.1038/leu.2015.284

[b56] Jawhar M. . Molecular profiling of myeloid progenitor cells in multi-mutated advanced systemic mastocytosis identifies KIT D816V as a distinct and late event. Leukemia 29, 1115–1122 (2015). 25567135 10.1038/leu.2015.4

[b57] Riera L. . Core binding factor acute myeloid leukaemia and c-KIT mutations. Oncol. Rep., doi: 10.3892/or.2013.2328 (2013). 23467883

[b58] Wang Y. . AML1-ETO and C-KIT mutation/overexpression in t(8;21) leukemia : Implication in stepwise leukemogenesis and response to Gleevec. Proc. Natl. Acad. Sci. USA 102, 1104–1109 (2005). 15650049 10.1073/pnas.0408831102 PMC545849

[b59] Bosbach B. . Imatinib resistance and microcytic erythrocytosis in a KitV558Δ;T669I/+gatekeeper-mutant mouse model of gastrointestinal stromal tumor. Proc. Natl. Acad. Sci. USA 109, E2276–83 (2012). 22652566 10.1073/pnas.1115240109 PMC3427109

[b60] Hasselbalch H. C. . High expression of carcinoembryonic antigen-related cell adhesion molecule (CEACAM) 6 and 8 in primary myelofibrosis. Leuk. Res. 35, 1330–1334 (2011). 21470677 10.1016/j.leukres.2011.03.013

[b61] Hasselbalch H. C. . Transcriptional Profiling of Whole Blood Identifies a Unique 5-Gene Signature for Myelofibrosis and Imminent Myelofibrosis Transformation. PLoS One 9, e85567 (2014). 24454890 10.1371/journal.pone.0085567 PMC3890316

[b62] Pérez C. . Aberrant DNA methylation profile of chronic and transformed classic Philadelphia-negative myeloproliferative neoplasms. Haematologica 98, 1414–1420 (2013). 23716560 10.3324/haematol.2013.084160 PMC3762098

[b63] Gordon H. & Sweets H. H. A Simple Method for the Silver Impregnation of Reticulum. Am. J. Pathol. 12, 545–552.1 (1936). 19970284 PMC1911089

[b64] Nyabi O. . Efficient mouse transgenesis using Gateway-compatible ROSA26 locus targeting vectors and F1 hybrid ES cells. Nucleic Acids Res. 37, e55 (2009). 19279185 10.1093/nar/gkp112 PMC2673446

[b65] Soriano P. Generalized lacZ expression with the ROSA26 Cre reporter strain. Nat. Genet. 21, 70–1 (1999). 9916792 10.1038/5007

