# Supplementary material for: The spleen microenvironment influences disease transformation in a mouse model of KITD816V-dependent myeloproliferative neoplasm
Source: Sci Rep. 2017 Jan 27;7:41427. doi: 10.1038/srep41427 (PMC5269732; doi:10.1038/srep41427)
Supplement: Supplementary Information [file srep41427-s1.pdf]

The spleen microenvironment influences disease transformation in a mouse model of KIT^{D816V}-dependent myeloproliferative neoplasm

Natalie Pelusi,[1#] Maike Kosanke,[2,3#] Tamara Riedt,[4] Corinna Rösseler,[2,3] Kristin Seré,[2,3] Jin Li,[4] Ines Gütgemann,[1] Martin Zenke,[2,3] Viktor Janzen,[4] and Hubert Schorle[1]*

Supplementary Information

Supplementary Methods

Phospho-flow cytometry

For flow cytometric analysis of phoshpo-proteins, BM cells were first starved for two hours in StemPro-34 medium. Cells were then used unstimulated for analysis of basal phospho-protein levels or stimulated for 10 minutes with either 100 ng/ml SCF or 50 ng/ml thrombopoietin. Stimulation was stopped with BD Phosflow Lyse/Fix Buffer for 10 minutes at 37 °C and cells were washed with BD Phosflow Perm/Wash Buffer I. Cells were then stained for 20 minutes at 4 °C with antibodies against surface markers (Lineage-cocktail 1, Sca1-PE, c-Kit-APC) and phosho-proteins (phospho-Akt or phospho-Erk1/2) diluted in BD Phosflow Perm/Wash Buffer I. After washing, cells were stained for 20 minutes at 4 °C with secondary antibodies (Streptavidin/Anti-Biotin eF450, Goat Anti-Rabbit IgG AF488). Finally, cells were washed and treated with BioLegend Fixation Buffer for 15 minutes at room temperature to extinguish endogenous GFP expression prior to detection.

Supplementary Table 1: Antibodies

Antigen	Fluorochrome	Clone	Dilution	Manufacturer
**Antibodies for flow cytometry**				
CD11b (Mac1A)	eFluor450	M1/70	1:500	eBioscience
CD11b (Mac1A)	Pacific Blue	M1/70	1:400	eBioscience
CD11b (Mac1A)	APC	M1/70	1:400	eBioscience
CD11c	PE	N418	1:400	eBioscience
CD115	APC	AFS98	1:400	eBioscience
CD115	PE	AFS98	1:400	eBioscience
CD127	APC-eFluor780	A7R34	1:100	eBioscience
CD127	PE-Cyanine5.5	A7R34	1:400	eBioscience
CD150/SLAM	Pacific Blue	TC15-12F12.2	1:100	BioLegend
CD150/SLAM	PE-Cyanine5.5	TC15-12F12.2	1:400	BioLegend
CD19	Pacific Blue	eBio1D3	1:400	eBioscience
CD3e	PE-Cyanine7	145-2C11	1:500	eBioscience
CD34	eFluor450	RAM34	1:200	eBioscience
CD34	APC	RAM34	1:400	BD Pharmingen
CD41	PE	MWReg30	1:400	BD Bioscience
CD44	PE	IM7	1:1000	BD Pharmingen
CD45	PE	30-F11	1:1000	BD Pharmingen
CD45	APC-Cyanine7	30-F11	1:400	eBioscience
CD45R/B220	PerCP	RA3-6B2	1:500	BD Pharmingen
B220	PerCP-Cyanine5.5	RA3-6B2	1:500	eBioscience
B220	PE-Cyanine5.5	RA3-6B2	1:400	eBioscience
B220	APC	RA3-6B2	1:400	eBioscience
CD48	APC-Cyanine7	HM48-1	1:700	BD Pharmingen
CD62L	Pacific Blue	MEL-14	1:400	eBioscience
CD71 (Tfrc)	APC	R17217	1:500	eBioscience
c-Kit	APC	2B8	1:100	eBioscience
c-Kit	PE-Cyanine7	2B8	1:200	BD Pharmingen
Goat Anti-Rabbit igG	Alexa Fluor 488		1:500	life technologies
Gr-1/Ly-6G	eFluor660	RB6-8C5	1:500	eBioscience
Gr-1	PE-Cyanine5.5	RB6-8C5	1:400	BD Bioscience
CD16/32 (FcγRII/III)	PE	93	1:1000	eBioscience
F4/80	Biotin		1:400	CALTAG-Laboratories
FcεRI	APC	MAR-1	1:400	BioLegend
Flt3	PE	A2F10	1:400	eBioscience
Ki67	FITC	SolA15	1:50	eBioscience
phospho-Akt (Ser473)	-	D9E	1:100	Cell Signaling
phospho-p44/42 (Erk1/2) (Thr202/Tyr204)	-		1:200	Cell Signaling
Sca1	PE	D7	1:200	eBioscience
Sca1	PE-Cyanine7	D7	1:300	eBioscience
Sca1	PE-Cyanine5.5	D7	1:400	eBioscience
Sca1	APC		1:80	MACS Miltenyi Biotec
SiglecH	PE-Cyanine5.5	eBio440c	1:400	eBioscience
TER-119	APC-Cyanine7	TER-119	1:500	eBioscience
Lineage-Cocktails	Biotin		1:50	
Streptavidin/Anti-Biotin	eF450		1:1000	eBioscience
Streptavidin/Anti-Biotin	PerCP		1:1000	eBioscience
Streptavidin/Anti-Biotin	PerCP Cyanine5.5		1:1000	eBioscience

Streptavidin/Anti-Biotin	Pacific Blue		1:400	eBioscience
Streptavidin/Anti-Biotin	PE-Cyanine7		1:400	eBioscience
Streptavidin/Anti-Biotin	PE		1:400	eBioscience
**Antibodies for Immunohistochemistry**				
KI67		SP6		Abcam
Caspase3 (cleaved)				Zytomed Systems
biotinylated secondary antibodies				DAKO

Supplementary Table 2: Marker profiles for flow cytometric analysis of hematopoietic cell populations

Cell Population	Tissue	Markers
Monocytes	peripheral blood (pB), spleen	CD45^{pos}CD11b^{pos}Gr-1^{neg}
Monocytes	bone marrow	CD45^{pos}Lin3^{neg}Gr1$^{pos/neg}$CD115^{pos}CD62L$^{neg/low}$CD11b^{pos}
Granulocytes	pB, spleen	CD45^{pos}Mac1^{pos}Gr-1^{pos}
Granulocytes	bone marrow	CD45^{pos}Lin3^{neg}Gr-1$^{pos/neg}$CD62L^{pos}
B-Cells	pB, spleen	CD45^{pos}CD11b^{neg}Gr1^{neg}B220^{pos}CD3^{neg}
B-Cells	bone marrow	CD45^{pos}Lin4^{neg}CD19^{pos}B220^{pos}
T-Cells	pB, spleen	CD45^{pos}Mac-1^{neg}Gr1^{neg}B220^{neg}CD3^{pos}
pDCs	bone marrow, spleen	CD45^{pos}Lin4^{neg}CD19^{neg}B220^{pos}CD11c^{pos}SiglecH^{pos}
cDCs	bone marrow, spleen	CD45^{pos}Lin4^{neg}CD19^{neg}B220^{neg}CD11c^{pos}
Mast cells	peritoneal cavity	CD45^{pos}B220^{neg}Kit^{pos}FcεRI^{pos}
Mast cells	skin	CD45^{pos}AquaZombie^{neg}CD3^{neg}Kit^{pos}FcεRI^{pos}
Erythroblasts	peripheral blood	CD45^{neg}CD71^{pos}Ter-119$^{pos/neg}$
CMPs	bone marrow, spleen	CD45^{pos}Lin2^{neg}Kit^{pos}Sca-1^{neg}CD34^{low}CD16/CD32^{low}
GMPs	bone marrow, spleen	CD45^{pos}Lin2^{neg}Kit^{pos}Sca-1^{neg}CD34^{low}CD16/CD32^{high}
MEPs	bone marrow, spleen	CD45^{pos}Lin2^{neg}Kit^{pos}Sca-1^{neg}CD34^{neg}CD16/CD32^{neg}
MDPs	bone marrow, spleen	CD45^{pos}Lin5^{neg}Kit^{pos}Flt3^{pos}CD115^{pos}
CDPs	bone marrow, spleen	CD45^{pos}Lin5^{neg}Kit^{int}Flt3^{pos}CD115^{pos}CD127^{neg}
MkPs	bone marrow, spleen	CD45^{pos}Lin2^{neg}Kit^{pos}Sca-1^{neg}CD150^{pos}CD41^{pos}
CLPs	bone marrow, spleen	CD45^{pos}Lin5^{neg}Kit^{int}Flt3^{pos}CD115^{neg}CD127^{pos}
HSCs	bone marrow, spleen	Lin1^{neg}Kit^{pos}Sca-1^{pos}CD48^{neg}CD150^{pos}
LT-HSCs	bone marrow, spleen, pB	CD45^{pos}Lin2^{neg}Kit^{pos}Sca-1^{pos}CD34^{neg}Flt3^{neg}
ST-HSCs	bone marrow, spleen, pB	CD45^{pos}Lin2^{neg}Kit^{pos}Sca-1^{pos}CD34^{pos}Flt3^{neg}
MPPs	bone marrow, spleen, pB	CD45^{pos}Lin2^{neg}Kit^{pos}Sca-1^{pos}CD34^{pos}Flt3^{pos}
**Lineage marker cocktails**		
Lineage cocktail 1 (Lin1)		B220; CD3; Ter119; CD11b; Gr-1
Lineage cocktail 2 (Lin2)		B220; CD3; CD8; Ter119; CD11c; CD11b; NK1.1; Gr1; MHCII; CD127; CD19
Lineage cocktail 3 (Lin3)		B220; CD3; Ter119; NK1.1; MHCII; CD19
Lineage cocktail 4 (Lin4)		CD3; Ter119; CD11b; NK1.1
Lineage cocktail 5 (Lin5)		B220; CD3; CD8; Ter119; CD11c; CD11b; NK1.1; Gr1; MHCII; CD19

Supplementary Table 3: Primer sequences for qRT-PCR

Target	Primer sequence
Bmi1 Forward	TGTGTCCTGTGTGGAGGGTA
Bmi1 Reverse	GGACAATACTTGCTGGTCTCCA
c-KIT Ex20 Forward (human)	GGGATGCAGATCCCCTAAA
c-KIT Ex21 Reverse (human)	GACAGAATTGATCCGCACAG
c-Myc Ex1 Forward	CTGGATTTCCTTTGGGCGTT
c-Myc Ex2 Reverse	TGGTGAAGTTCACGTTGAGGG
c-Myb Ex9 Forward	TCACCAGCAAGGTGCATGAT
c-Myb Ex10 Reverse	GGTAGGTGCATCTAAGCCCG
CopGFP Forward	CTACCACTTCGGCACCTACC
CopGFP Reverse	GGTGAAGATCACGCTGTCCT
Foxo3a Forward	GATAAGGGCGACAGCAACAG
Foxo3a Reverse	CTTCATTCTGAACGCGCATGA
GATA-2 Ex3 Forward	CCTCCAGCTTCACCCCTAAG
GATA-2 Ex4 Reverse	ACAGGCATTGCACAGGTAGT
Gusb Forward	GGCTGGTGACCTACTGGATTT
Gusb Reverse	TTGGCACTGGGAACCTGAAGT
Hoxb4 Forward	TACCCCTGGATGCGCAAAG
Hoxb4 Reverse	CGCGTCAGGTAGCGATTGT
mKit Ex20 Forward (murine)	AGAAGCAGATCTCGGACAGC
mKit Ex21/UTR Reverse (murine)	CGTAAAGGCGGAATCACAGT
Runx1 Forward	CCAGCCTCTCTGCAGAACTTT
Runx1 Reverse	TGGACGGCAGAGTAGGGAAC
Sdha Forward	GCTCCTGCCTCTGTGGTTGA
Sdha Reverse	AGCAACACCGATGAGCCTG
Tal1 Ex2 Forward	CGGAGGAGTGGAGGTCCTAA
Tal1 Ex3 Reverse	CCTCTAGTTGCGGGTCACTG

Supplementary Figure Legends

Supplementary Figure S1: Induction of KIT^{D816V} expression in HSC-SCL:KIT^{D816V} mice. (a) Unfractionated cells and indicated cell populations purified by flow cytometric cell sorting from BM and spleen were analyzed for expression of endogenous Kit and the KIT^{D816V} transgene by quantitative real-time PCR (qRT-PCR). Cells were isolated from HSC-SCL:KIT^{D816V} and control mice 4 to 6 weeks after TX injection. Data are presented as relative expression normalized to Gusb and Sdha. Unfractionated BM - Control: N=6. HSC-SCL:KIT^{D816V}: N=5. Unfractionated spleen - Control: N=3. HSC-SCL:KIT^{D816V}: N=3. EryB - Control: N=3. HSC-SCL:KIT^{D816V}: N=3. $Lin^{neg}Kit^{pos}$ - Control: N=3. HSC-SCL:KIT^{D816V}: N=3. $Lin^{neg}Kit^{neg}$ - Control: N=3. HSC-SCL:KIT^{D816V}: N=2. EryB: Erythroblasts. (b) GFP^{pos} and GFP^{neg} cell populations were purified by flow cytometric cell sorting from HSC-SCL:KIT^{D816V} BM and spleen 6 weeks after TX injection and analyzed for KIT^{D816V} expression by qRT-PCR. In GFP^{neg} spleen cells, KIT^{D816V} transcript was also detected. This is presumably due to the high fraction of terminally differentiated red cells, that were shown to loose the GFP signal (orthochromatic erythroblasts, reticulocytes; compare Supplementary Figure 3G). We hypothesize that in these cells the GFP protein may be rapidly degraded while the transcript is more stable. Similar detection of the copepod GFP (CopGFP) transcript in GFP^{neg} spleen cells supports this assumption. Data are shown as relative expression normalized to Gusb and Sdha. HSC-SCL:KIT^{D816V}: N=2. Data are presented as mean±standard deviation. P-values were determined using one-way ANOVA with Bonferroni post-tests.

Supplementary Figure S2: Analysis of pB from HSC-SCL:KIT^{D816V} and control mice. (a) Indicated pB parameters of HSC-SCL:KIT^{D816V} and control mice were analyzed 4, 8, 10 and 18 weeks after TX injection using a hematology analyzer. 4W - Control: N=20. HSC-SCL:KIT^{D816V}: N=14. 8W - Control: N=17. HSC-SCL:KIT^{D816V}: N=9. 10W - Control: N=7. HSC-SCL:KIT^{D816V}: N=6. 18W - Control: N=4. HSC-SCL:KIT^{D816V}: N=2. MCH: mean corpuscular hemoglobin. MCHC: mean corpuscular hemoglobin concentration. MPV: mean platelet volume. RDW: red blood cell distribution width. (b) Nucleated pB cells were analyzed at indicated time points after TX-treatment for expression of CD45 and markers for lineage committed blood cells via flow cytometry. The WBC was used to calculate the total number of cells per unit of blood volume. 4W - Control: N=18. HSC-SCL:KIT^{D816V}: N=13. 10W - Control: N=4. HSC-SCL:KIT^{D816V}: N=4. EryB: Erythroblasts. Gran: Granulocytes. Mono: Monocytes. (c) Representative flow cytometric panels illustrating the gating strategies for lineage analyses of pB cells. (d) The different granulocyte populations in pB of HSC-SCL:KIT^{D816V} and control mice were 4 weeks after TX injection using a hematology analyzer. Notably, total and relative numbers both were found elevated for basophils. Control: N=7. HSC-SCL:KIT^{D816V}: N=7. (e) The reticulocyte frequency in pB from HSC-SCL:KIT^{D816V} and control mice was determined by flow cytometry 5-7 weeks after TX injection by staining with thiazol orange. Control: N=6. HSC-SCL:KIT^{D816V} : N=5. Blood smears of HSC-SCL:KIT^{D816V} and control mice were subjected to May-Grünwald-Giemsa staining. (f) Serum thrombopoietin levels were determined 4 weeks after induction using ELISA. Control: N=6. HSC-SCL:KIT^{D816V}: N=6. Data are presented as mean±standard deviation. P-values were determined using two-tailed, unpaired Student's t-test.

Supplementary Figure S3: Analysis of bone marrow and spleen erythropoiesis in HSC-SCL:KIT^{D816V} and control mice. (a) BM megakaryocytes were quantified on HE stained BM paraffin sections. Megakaryocytes from three randomly chosen fields of vision were counted (20x objective) and the fold change to controls was determined for HSC-SCL-Cre:KIT^{D816V} mice. Control: N=6. HSC-SCL:KIT^{D816V} : N=4. (b) BM paraffin sections from control and HSC-SCL-Cre:KIT^{D816V} mice were subjected to immunohistochemical staining for Ki67 and active Caspase3. (c) Flow cytometric dot plots of representative samples illustrating the gating strategy for analysis of BM erythropoiesis beginning from the proerythroblast stage. Black numbers: cell frequency in relation to parent gate. Red labels: successive stages of erythroid development. Left graph: Absolute cell numbers of erythroid subpopulations in BM. Control: N=7. HSC-SCL:KIT^{D816V} : N=5. Right graph: Frequency of GFP-positive cells within indicated populations. HSC-SCL:KIT^{D816V}: N=8. (d) CFU-E assays were performed with BM cells. Control: N=4. HSC-SCL:KIT^{D816V}: N=4. (e-f) HE staining (e) and immunohistological CD3 staining (f) on paraffin sections from HSC-SCL:KIT^{D816V} and control spleen. Only few CD3-positive cells were found dispersed across the spleen tissue of HSC-SCL:KIT^{D816V} mice. (g) Flow cytometric gating for analysis of splenic erythropoiesis is illustrated for representative samples. Graphs show cell frequencies of erythroid subpopulations and the frequency of GFP-positive cells within indicated populations for HSC-SCL:KIT^{D816V} mice. Control: N=10. HSC-SCL:KIT^{D816V}: N=8. Data are presented as mean±standard deviation. P-values were determined using two-tailed, unpaired Student’s t-test.

Supplementary Figure S4: KIT^{D816V} regulates proliferation in hematopoietic stem cell compartments and mediates mobilization of stem cells and progenitors from BM to spleen. (a) HSC-SCL:KIT^{D816V} and control mice were subjected to TX-treatment and analyzed after 7-10 weeks. Graphs show quantification of stem cell populations in absolute cell numbers for BM and frequencies of CD45-positive cells for spleen. BM - Control: N=7. HSC-SCL:KIT^{D816V}: N=5. Spleen - Control: N=9. HSC-SCL:KIT^{D816V}: N=7. (b) The Lin^{neg}Kit^{pos} cell population was purified from HSC-SCL:KIT^{D816V} and control BM 4 to 6 weeks after TX injection by flow cytometric cell sorting and analyzed for expression of indicated genes by quantitative real-time PCR. Data are presented as relative expression normalized to Gusb and Sdha. Control: N=3. HSC-SCL:KIT^{D816V}: N=3. (c) Representative flow cytometric density plots illustrating the analysis of the cell cycle status of indicated BM stem cell populations by combined Ki67/DAPI staining shown in main Figure 3B. (d) BM LK and LSK populations of HSC-SCL:KIT^{D816V} and control mice were analyzed by phospho-flow cytometry in the second week after TX-treatment. Cells were used unstimulated or stimulated for 10 minutes with indicated concentrations of SCF or thrombopoietin. No differences in basal phosphorylation or reaction to cytokine stimulation were detected, as illustrated by representative flow cytometric panels. Control: N=2. HSC-SCL:KIT^{D816V}: N=2. (e-f) HSC-SCL:KIT^{D816V} and control mice were subjected to TX-treatment and BM and spleen cells were analyzed by flow cytometry 7 weeks after induction. Control: N=3. HSC-SCL:KIT^{D816V}: N=3. (e) Absolute numbers of LT-HSCs, ST-HSCs and MPPs. (f) Absolute cell numbers of indicated progenitor cell populations. (g) The marker profile for analysis of LT-HSCs, ST-HSCs, MPPs and progenitor populations is illustrated by

flow cytometric gating panels. Data are presented as mean±standard deviation. P-values were determined using two-tailed, unpaired Student's t-test.

Supplementary Figure S5: Analysis of GFP expression in KIT^{D816V} mutant mice. (a) Flow cytometric analysis of Kit surface expression in HSC-SCL:KIT^{D816V} BM cell populations that are positive or negative for the GFP reporter that is coexpressed with KIT^{D816V}. Representative plots show similar levels of Kit on the cell surface of GFP-positive and GFP-negative populations, as the mutant receptor has been shown to localize to subcellular compartments.[3] The extracellular part of the hybrid KIT^{D816V} protein consists of the murine sequence, therefore, the antibody used for analysis recognizes the endogenous as well as the ectopically expressed receptor. Successful coexpression of GFP and KIT^{D816V} on protein level in R26-LSL-KIT^{D816V}. mice has been validated before.[4] (b) The frequency of GFP-positive cells in indicated cell populations of HSC-SCL:KIT^{D816V} mice is illustrated. Representative flow cytometric panels of the GFP-analysis are shown. BM - HSC-SCL:KIT^{D816V}: N=5. Spleen - HSC-SCL:KIT^{D816V}: N=7. (c) Diagram illustrating the frequencies of GFP-positive cells throughout cell populations examined 6-10 weeks after TX administration. (d-f) HSC-SCL:KIT^{D816V} mice were subjected to TX-treatment and the frequency of GFP-positive cells in indicated cell populations was analyzed after 2, 4 and 6 weeks. 2W - HSC-SCL:KIT^{D816V}: N=3. 4W - HSC-SCL:KIT^{D816V}: N=2. 6W - HSC-SCL:KIT^{D816V}: N=2. (d) Analysis of LT-HSC, ST-HSC and MPP populations. (e) Analysis of progenitor populations. (f) Analysis of mature B-cell, monocyte and granulocyte populations. (g) Erythoid populations from BM of HSC-SCL:KIT^{D816V} mice were analyzed by flow cytometry for the frequency of GFP-positive cells directly after TX-induction. Graph illustrates the frequency of GFP-positive cells within indicated cell populations. HSC-SCL:KIT^{D816V}: N=3. Data are presented as mean±standard deviation. P-values were determined using two-tailed, unpaired Student's t-test.

Supplementary Figure S6: Analysis of mature BM blood cell populations. (a) HSC-SCL:KIT^{D816V} and control mice were subjected to TX-treatment. 7 weeks after KIT^{D816V} induction BM was analyzed for indicated cell populations via flow cytometry. Absolute cell numbers per femur are shown. Flow cytometric panels illustrate the gating strategy for each cell population. Control: N=3. HSC-SCL:KIT^{D816V}: N=3. DC: Dendritic cell. cDC: Conventional DC. pDC: Plasmacytoid DC. (b) Flow cytometric gating strategies for peritoneal and skin mast cell populations that are quantified in main Figure 6D are shown. Pictures show toluidine blue staining for mast cells on skin sections, revealing no difference between control and HSC-SCL:KIT^{D816V} mice. Data are presented as mean±standard deviation. P-values were determined using two-tailed, unpaired Student's t-test.

Supplementary Figure S7: Analysis of extramedullary hematopoiesis after BM transplantation. Whole BM cells from HSC-SCL:KIT^{D816V} and control mice were used for BM transplantation 6 weeks after KIT^{D816V}-induction. Recipients were analyzed 3 and 6 weeks post-transplantation. Control donors: N=1. HSC-SCL:KIT^{D816V} donors: N=3. 3W - Control recipients: N=1. HSC-SCL:KIT^{D816V} recipients: N=3. 6W - Control recipients: N=2 for (a); N=1 for (b-e). HSC-SCL:KIT^{D816V} recipients: N=5. (a) The WBC was analyzed with a hematology analyzer. (b) The spleen weight of donors and recipients at indicated time points is depicted. (c) Spleen cells were analyzed via flow cytometry for erythroblast (EryB) markers. Absolute cell numbers per organ are shown. (d-e) Quantification of splenic HSC/HPC populations. (d) Relative frequencies shown for 3W recipients. (e) Absolute cell numbers per organ. Data are presented as mean±standard deviation.

Supplementary Figure S8: Analysis of SplE HSC-SCL:KIT^{D816V} mice. HSC-SCL:KIT^{D816V} and control mice were subjected to SplE, followed by TX-treatment. (a) Indicated pB parameters were analyzed 4, 10 and 18 weeks after KIT^{D816V} induction. SplE Control: N=4. SplE HSC-SCL:KIT^{D816V}: N=3. (b) Reticulocytes in pB samples from SplE HSC-SCL:KIT^{D816V} and control mice were stained with thiazol orange and analyzed by flow cytometry 10 weeks after induction. SplE Control: N=5. SplE HSC-SCL:KIT^{D816V}: N=4. (c) Nucleated pB cells were analyzed 10 and 18 weeks after TX-treatment for expression of CD45 and markers for lineage committed blood cells via flow cytometry. The WBC was used to calculate the total number of cells per unit of blood volume. SplE Control: N=5. SplE HSC-SCL:KIT^{D816V}: N=4. EryB: Erythroblasts. Gran: Granulocytes. Mono: Monocytes. (d) BM was analyzed 10 weeks after KIT^{D816V} induction. Left bar graph shows total cellularity. Right bar graph shows percentage of CD45-positive cells. For HSC-SCL:KIT^{D816V} mice, the frequency of GFP-positive cells within each population is indicated. SplE Control: N=4. SplE HSC-SCL:KIT^{D816V}: N=3. (e) BM megakaryocytes were quantified on HE stained BM paraffin sections. Megakaryocytes from three randomly chosen fields of vision were counted (20x objective) and the fold change to controls was determined for SplE HSC-SCL-Cre:KIT^{D816V} mice. SplE Control: N=3. SplE HSC-SCL:KIT^{D816V} : N=2. (f) BM paraffin sections were subjected to Prussian Blue staining for hemosiderin. (g-j) Flow cytometric analysis of BM and liver erythropoiesis. (g) Analysis of BM erythropoiesis 10 weeks after KIT^{D816V} induction. Left: EryB frequency. Right: Fold change in frequency of erythroid subpopulations for HSC-SCL:KIT^{D816V} mice in relation to controls; Log10 scale. (h) Analysis of BM erythropoiesis 18 weeks after KIT^{D816V} induction. Dot plots illustrate flow cytometric gating for representative samples. Graph shows fold change in frequency of erythroid subpopulations for HSC-SCL:KIT^{D816V} mice in relation to controls; Log10 scale. (i) Absolute cell numbers of

erythroid BM subpopulations (left graphs) and frequencies of GFP-positive cells within indicated populations (right graphs) for 10 and 18 week analyses. SplE Control: N=4. SplE HSC-SCL:KIT^{D816V}: N=3. (j) Liver erythropoiesis analyzed 18 weeks after KIT^{D816V} induction. Left: EryB frequency. Middle: Fold change in frequency of erythroid subpopulations for HSC-SCL:KIT^{D816V} mice in relation to controls; Log10 scale. Right: Frequency of GFP-positive cells within indicated populations. SplE Control: N=4. SplE HSC-SCL:KIT^{D816V}: N=3. ProE: Proerythroblasts; BasoE: Basophilic EryB; PolyE: Polychromatic EryB; OrthoE: Orthochromatic EryB; Retic: Reticulocytes. Data are presented as mean±standard deviation. P-values were determined using two-tailed, unpaired Student's t-test.

Supplementary Figure S9: Stem cell analysis and histological reticulin and Ki67 staining in SplE KIT^{D816V} mutants. (a-b) HSC-SCL:KIT^{D816V} and control mice were subjected to SplE and TX-treatment and indicated BM cell populations were analyzed after 10 and 18 weeks. SplE Control: N=4 SplE HSC-SCL:KIT^{D816V}: N=3. (a) Graphs show quantification of stem cell populations in absolute cell numbers. (b) The frequency of GFP-positive cells in indicated cell populations of SplE HSC-SCL:KIT^{D816V} mice is illustrated. (c) BM LK and LSK populations of SplE HSC-SCL:KIT^{D816V} and SplE control mice were analyzed 10 weeks after TX-treatment by phospho-flow cytometry. Cells were used unstimulated or stimulated for 10 minutes with indicated concentrations of SCF or thrombopoietin. No differences in basal phosphorylation or the reaction to cytokine stimulation were detected, as illustrated by representative flow cytometric panels. SplE Control: N=2. SplE HSC-SCL:KIT^{D816V}: N=2. (d) BM sections from HSC-SCL:KIT^{D816V} and control mice without SplE (left) or with SplE (right) were stained for reticulin fibers 10 or 18 weeks after TX-treatment. In 10 weeks samples of SplE HSC-SCL:KIT^{D816V} mice focal networks of reticulin fibers were found (MF-0 according to the European Consensus fibrosis grading). (e) Spleen sections from HSC-SCL:KIT^{D816V} and control mice without SplE were stained for reticulin fibers 18 weeks after TX-treatment. (f) HSC-SCL:KIT^{D816V} and control mice were subjected to SplE and TX-treatment. BM paraffin sections were subjected to immunohistological staining against Ki67. 18 weeks KIT^{D816V} induction. Data are presented as mean±standard deviation. P-values were determined using two-tailed, unpaired Student's t-test.

References

1 Nyabi O, Naessens M, Haigh K, Gembarska A, Goossens S, Maetens M, *et al.* Efficient mouse transgenesis using Gateway-compatible ROSA26 locus targeting vectors and F1 hybrid ES cells. *Nucleic Acids Res* 2009; **37**: e55.

2 Soriano P. Generalized lacZ expression with the ROSA26 Cre reporter strain. *Nat Genet* 1999; **21**: 70–1.

3 Xiang Z, Kreisel F, Cain J, Colson A, Tomasson MH. Neoplasia driven by mutant c-KIT is mediated by intracellular, not plasma membrane, receptor signaling. *Mol Cell Biol* 2007; **27**: 267–82.

4 Haas N, Riedt T, Labbaf Z, Baßler K, Gergis D, Fröhlich H, *et al.* Kit transduced signals counteract erythroid maturation by MAPK-dependent modulation of erythropoietin signaling and apoptosis induction in mouse fetal liver. *Cell Death Differ* 2015; **22**: 790–800.

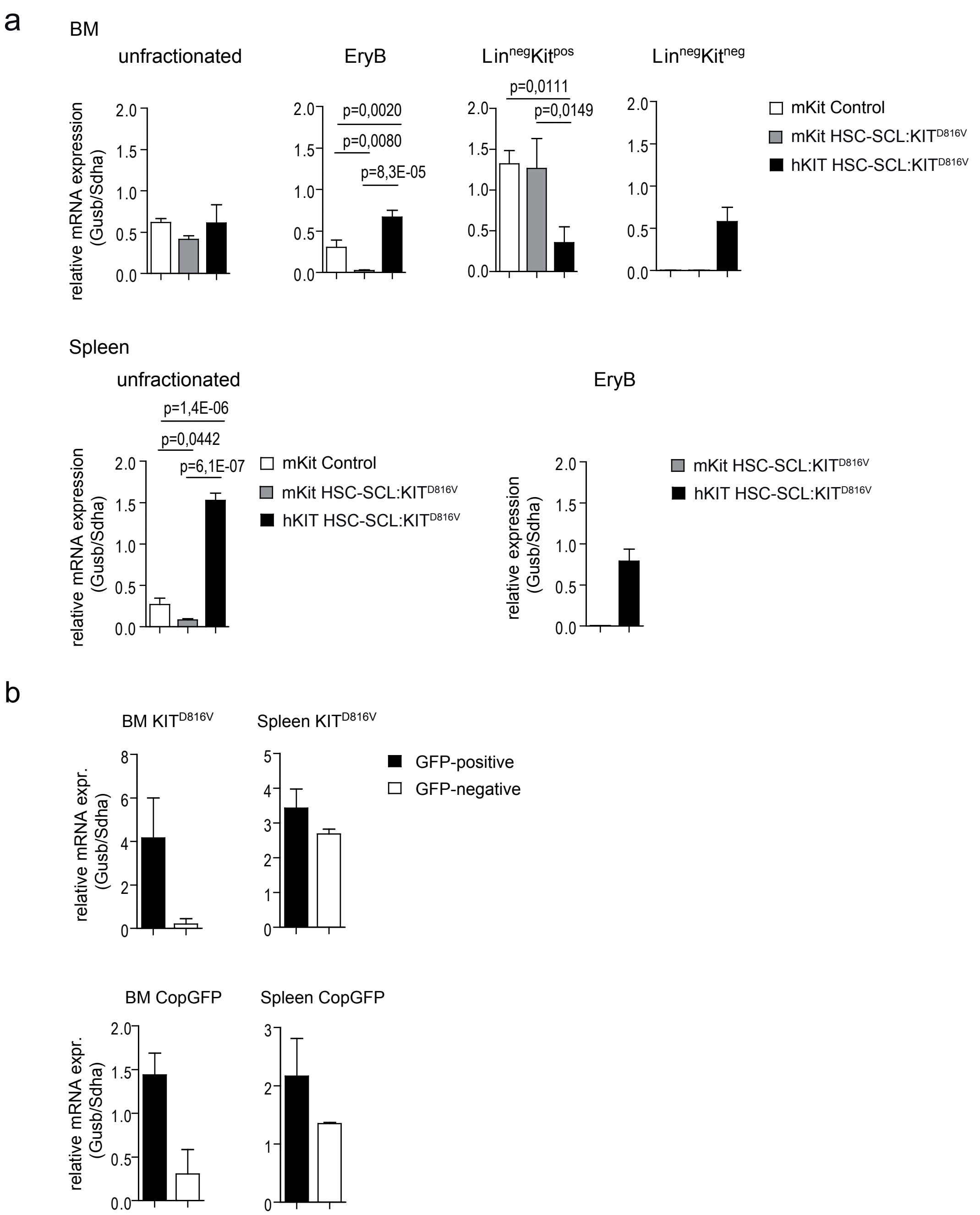
a
BM
unfractionated
EryB
Lin^{neg}Kit^{pos}
Lin^{neg}Kit^{neg}
relative mRNA expression (Gusb/Sdha)
2.0
1.5
1.0
0.5
0.0
p=0,0020
p=0,0080
p=8,3E-05
p=0,0111
p=0,0149
mKit Control
mKit HSC-SCL:KIT^{D816V}
hKIT HSC-SCL:KIT^{D816V}
Spleen
unfractionated
EryB
p=1,4E-06
p=0,0442
p=6,1E-07
relative expression (Gusb/Sdha)
b
BM KIT^{D816V}
Spleen KIT^{D816V}
relative mRNA expr. (Gusb/Sdha)
GFP-positive
GFP-negative
BM CopGFP
Spleen CopGFP

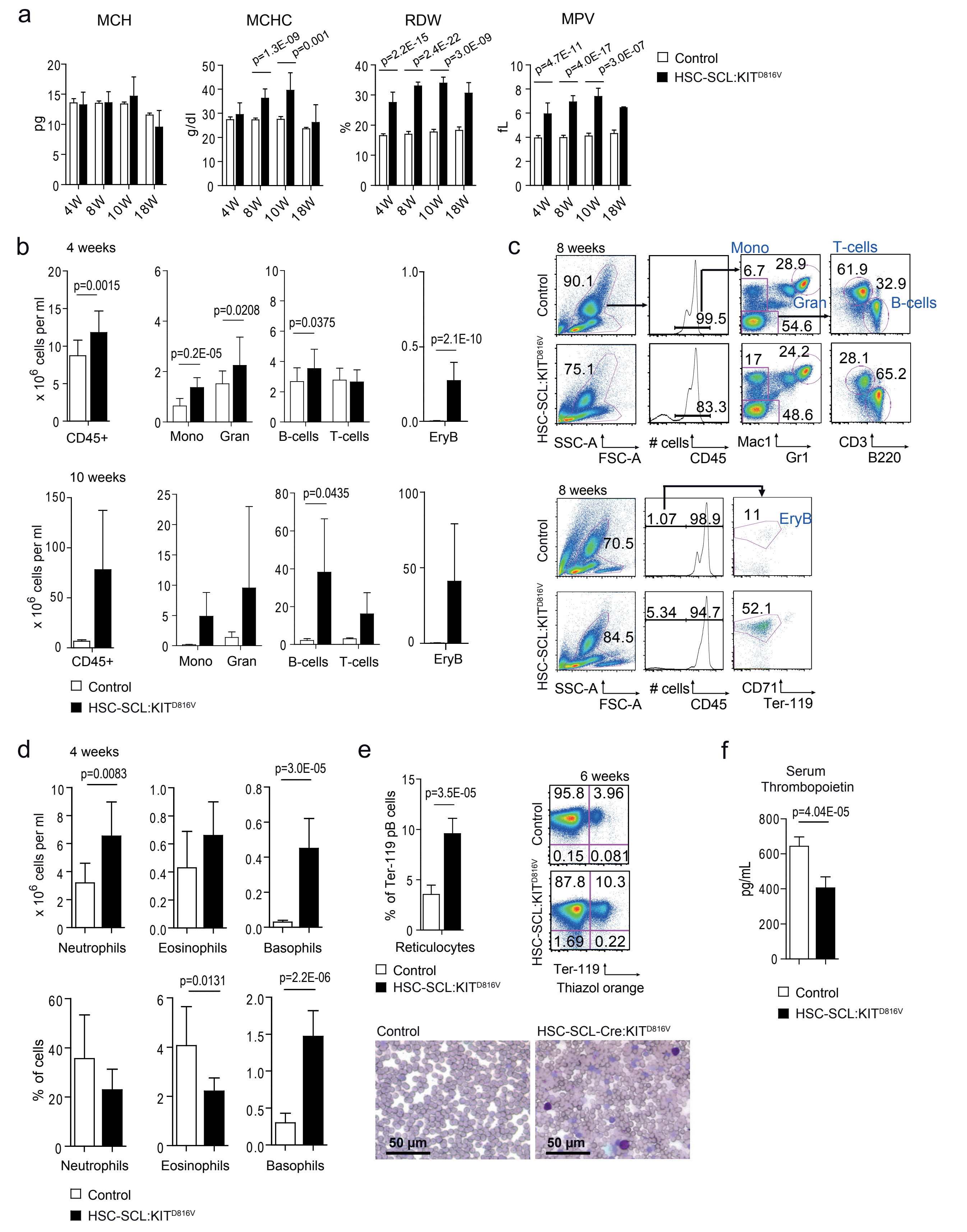
a
MCH
MCHC
RDW
MPV
pg
g/dl
%
fL
4W
8W
10W
18W
p=1.3E-09
p=0.001
p=2.2E-15
p=2.4E-22
p=3.0E-09
p=4.7E-11
p=4.0E-17
p=3.0E-07
Control
HSC-SCL:KIT^{D816V}
b
4 weeks
x 10^6 cells per ml
p=0.0015
p=0.2E-05
p=0.0208
p=0.0375
p=2.1E-10
CD45+
Mono
Gran
B-cells
T-cells
EryB
10 weeks
p=0.0435
Control
HSC-SCL:KIT^{D816V}
c
8 weeks
Control
HSC-SCL:KIT^{D816V}
90.1
99.5
6.7
28.9
54.6
61.9
32.9
Mono
Gran
T-cells
B-cells
75.1
83.3
17
24.2
48.6
28.1
65.2
SSC-A
FSC-A
cells
CD45
Mac1
Gr1
CD3
B220
8 weeks
70.5
1.07
98.9
11
EryB
84.5
5.34
94.7
52.1
CD71
Ter-119
d
4 weeks
x 10^6 cells per ml
p=0.0083
p=3.0E-05
Neutrophils
Eosinophils
Basophils
% of cells
p=0.0131
p=2.2E-06
Control
HSC-SCL:KIT^{D816V}
e
% of Ter-119 pB cells
p=3.5E-05
Reticulocytes
Control
HSC-SCL:KIT^{D816V}
6 weeks
95.8
3.96
0.15
0.081
87.8
10.3
1.69
0.22
Ter-119
Thiazol orange
Control
HSC-SCL-Cre:KIT^{D816V}
50 µm
50 µm
f
Serum Thrombopoietin
pg/mL
p=4.04E-05
Control
HSC-SCL:KIT^{D816V}

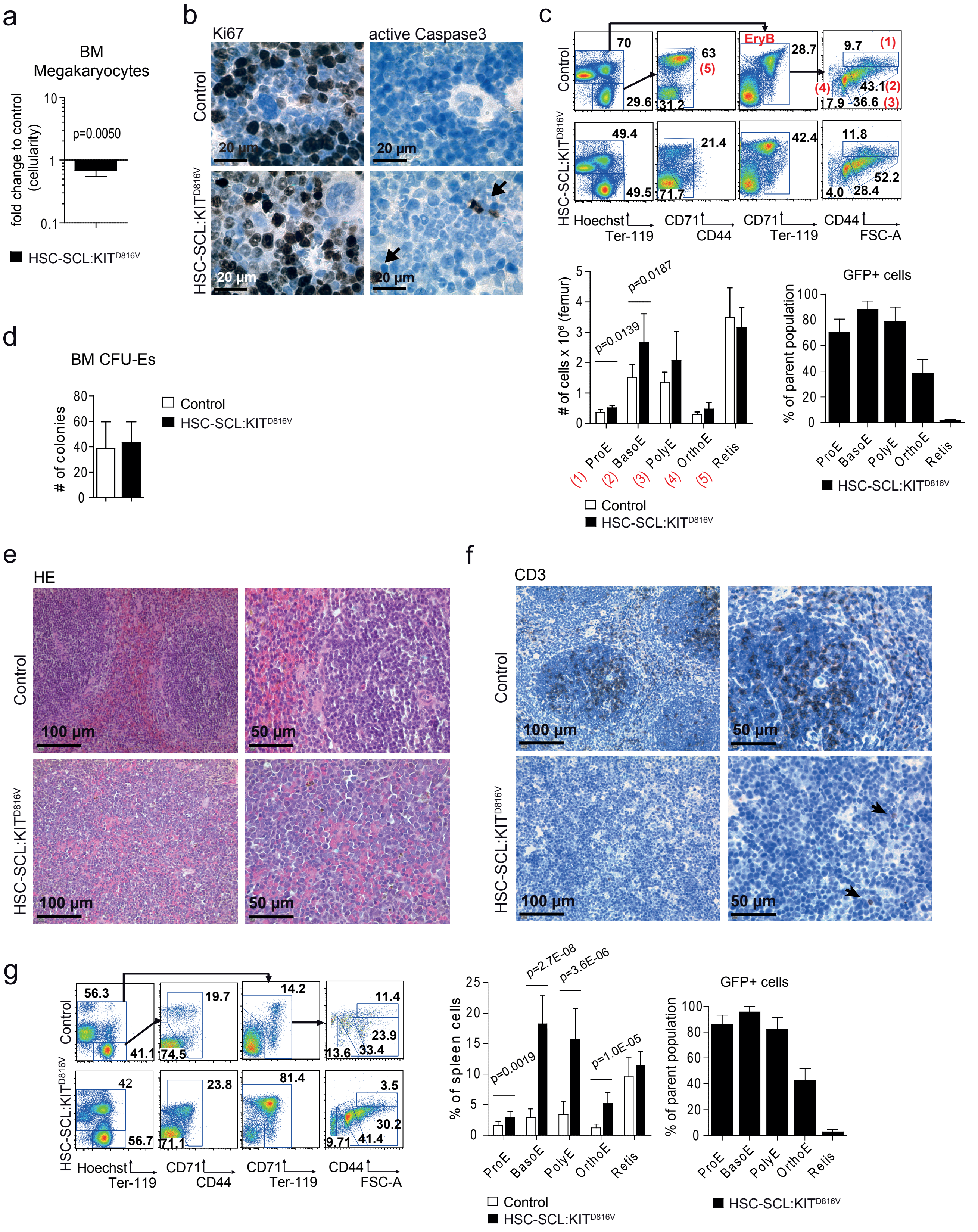
a
BM Megakaryocytes
fold change to control (cellularity)
p=0.0050
HSC-SCL:KIT D816V
b
Ki67
active Caspase3
Control
HSC-SCL:KIT D816V
20 µm
c
Control
HSC-SCL:KIT D816V
EryB
Hoechst
Ter-119
CD71
CD44
CD44
FSC-A
# of cells x 10⁶ (femur)
p=0.0139
p=0.0187
ProE
BasoE
PolyE
OrthoE
Retis
GFP+ cells
% of parent population
Control
HSC-SCL:KIT D816V
d
BM CFU-Es
# of colonies
Control
HSC-SCL:KIT D816V
e
HE
Control
HSC-SCL:KIT D816V
100 µm
50 µm
f
CD3
Control
HSC-SCL:KIT D816V
100 µm
50 µm
g
Control
HSC-SCL:KIT D816V
Hoechst
Ter-119
CD71
CD44
CD44
FSC-A
% of spleen cells
p=0.0019
p=2.7E-08
p=3.6E-06
p=1.0E-05
ProE
BasoE
PolyE
OrthoE
Retis
GFP+ cells
% of parent population
Control
HSC-SCL:KIT D816V

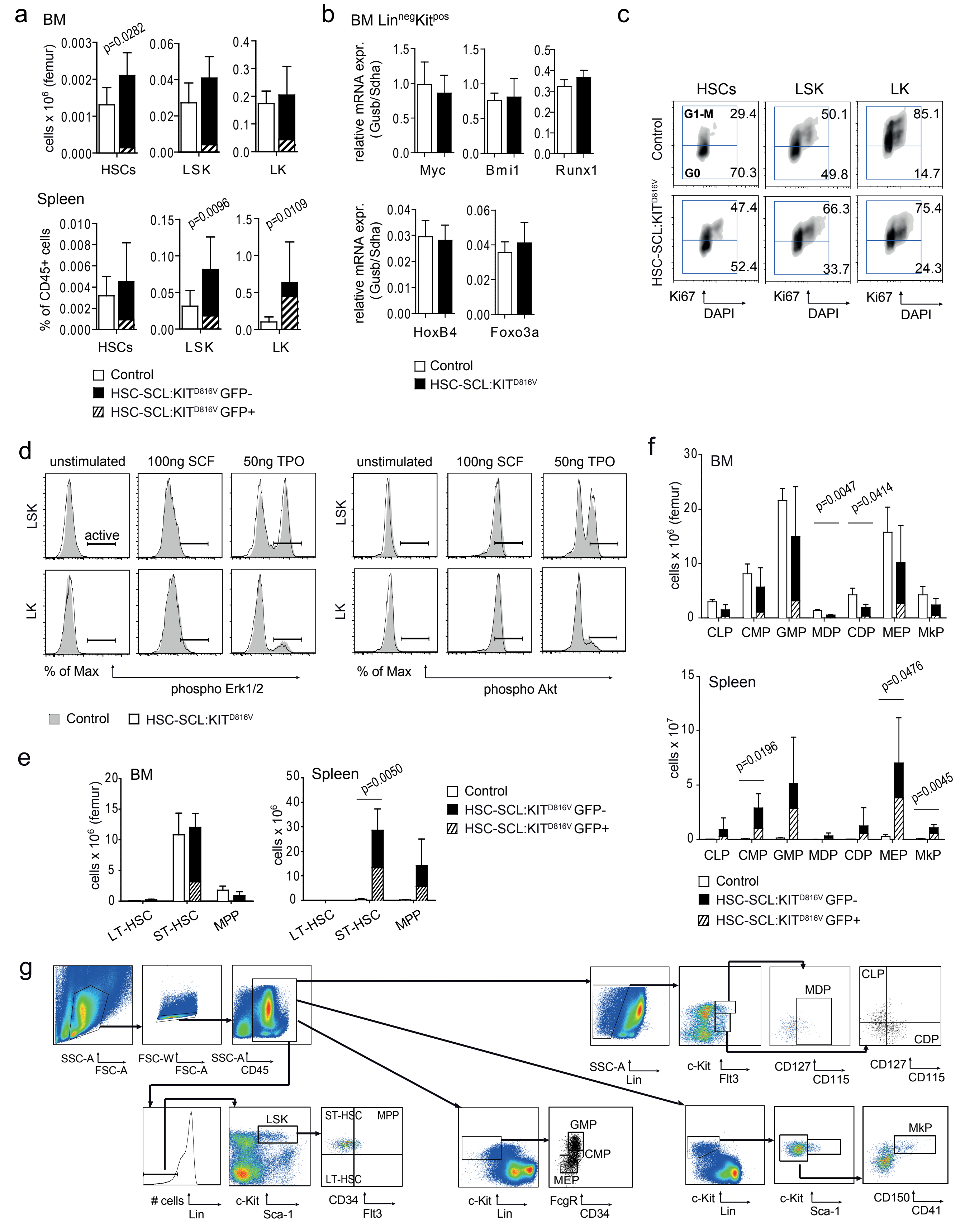
a
BM
p=0.0282
cells x 10^6 (femur)
HSCs
LSK
LK
Spleen
p=0.0096
p=0.0109
% of CD45+ cells
Control
HSC-SCL:KIT^D816V GFP-
HSC-SCL:KIT^D816V GFP+
b
BM Lin^neg Kit^pos
relative mRNA expr. (Gusb/Sdha)
Myc
Bmi1
Runx1
HoxB4
Foxo3a
Control
HSC-SCL:KIT^D816V
c
HSCs
LSK
LK
Control
HSC-SCL:KIT^D816V
G1-M
G0
29.4
70.3
50.1
49.8
85.1
14.7
47.4
52.4
66.3
33.7
75.4
24.3
Ki67
DAPI
d
unstimulated
100ng SCF
50ng TPO
LSK
LK
active
% of Max
phospho Erk1/2
phospho Akt
Control
HSC-SCL:KIT^D816V
e
BM
Spleen
p=0.0050
cells x 10^6 (femur)
cells x 10^6
LT-HSC
ST-HSC
MPP
Control
HSC-SCL:KIT^D816V GFP-
HSC-SCL:KIT^D816V GFP+
f
BM
p=0.0047
p=0.0414
cells x 10^6 (femur)
CLP
CMP
GMP
MDP
CDP
MEP
MkP
Spleen
p=0.0476
p=0.0196
p=0.0045
cells x 10^7
Control
HSC-SCL:KIT^D816V GFP-
HSC-SCL:KIT^D816V GFP+
g
SSC-A
FSC-A
FSC-W
FSC-A
SSC-A
CD45
SSC-A
Lin
c-Kit
Flt3
MDP
CD127
CD115
CLP
CDP
CD127
CD115
cells
Lin
LSK
c-Kit
Sca-1
ST-HSC
MPP
LT-HSC
CD34
Flt3
c-Kit
Lin
GMP
CMP
MEP
FcgR
CD34
c-Kit
Lin
c-Kit
Sca-1
MkP
CD150
CD41

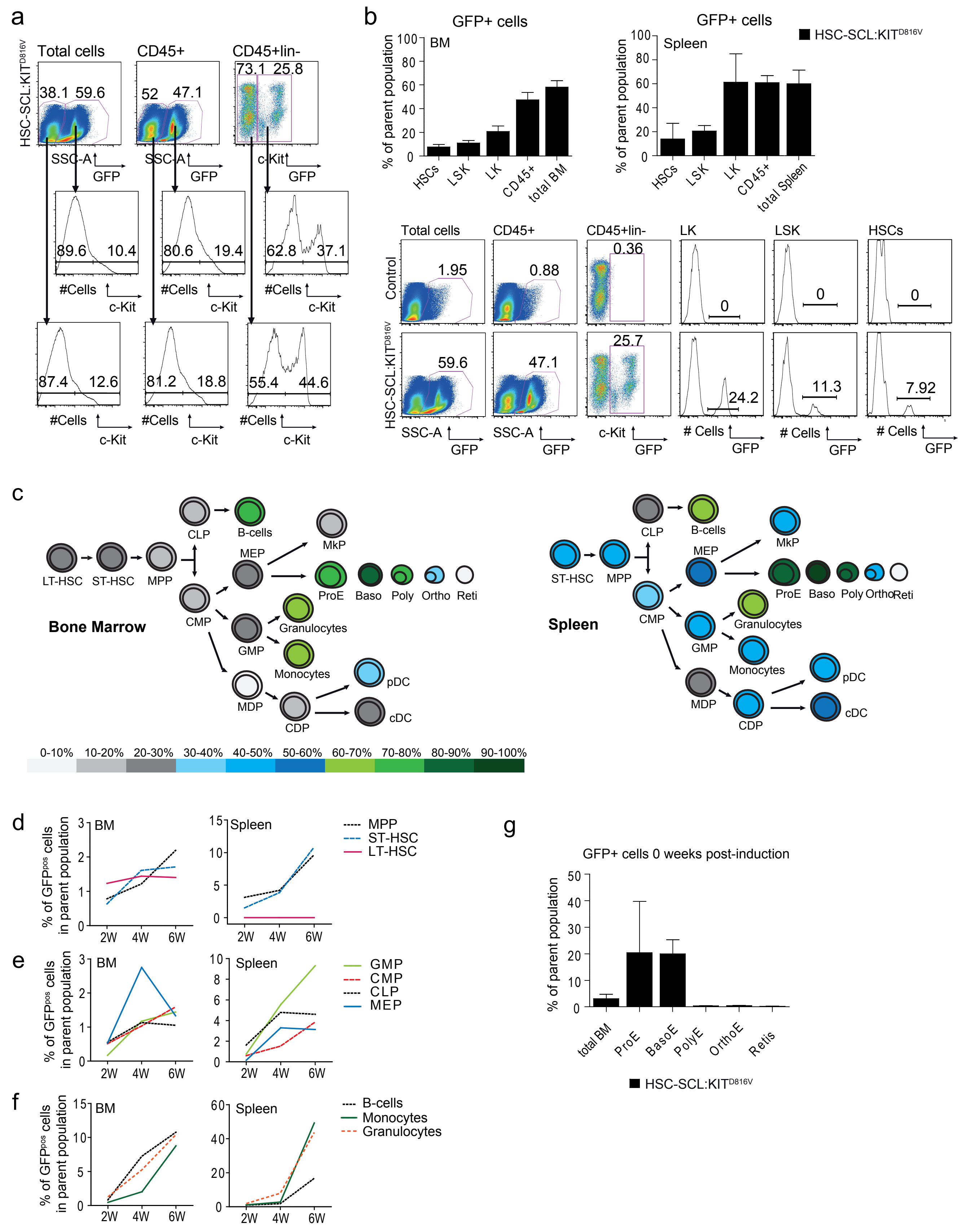
a
Total cells
CD45+
CD45+lin-
HSC-SCL:KIT D816V
SSC-A
GFP
c-Kit
#Cells
b
GFP+ cells
BM
Spleen
HSC-SCL:KIT D816V
% of parent population
HSCs
LSK
LK
CD45+
total BM
total Spleen
Control
LSK
HSCs
Cells
c
LT-HSC
ST-HSC
MPP
CLP
B-cells
MEP
MkP
CMP
ProE
Baso
Poly
Ortho
Reti
GMP
Granulocytes
Monocytes
MDP
CDP
pDC
cDC
Bone Marrow
Spleen
0-10%
10-20%
20-30%
30-40%
40-50%
50-60%
60-70%
70-80%
80-90%
90-100%
d
e
f
% of GFPpos cells in parent population
2W
4W
6W
MPP
ST-HSC
LT-HSC
GMP
CMP
CLP
MEP
B-cells
Monocytes
Granulocytes
g
GFP+ cells 0 weeks post-induction
total BM
ProE
BasoE
PolyE
OrthoE
Retis

a

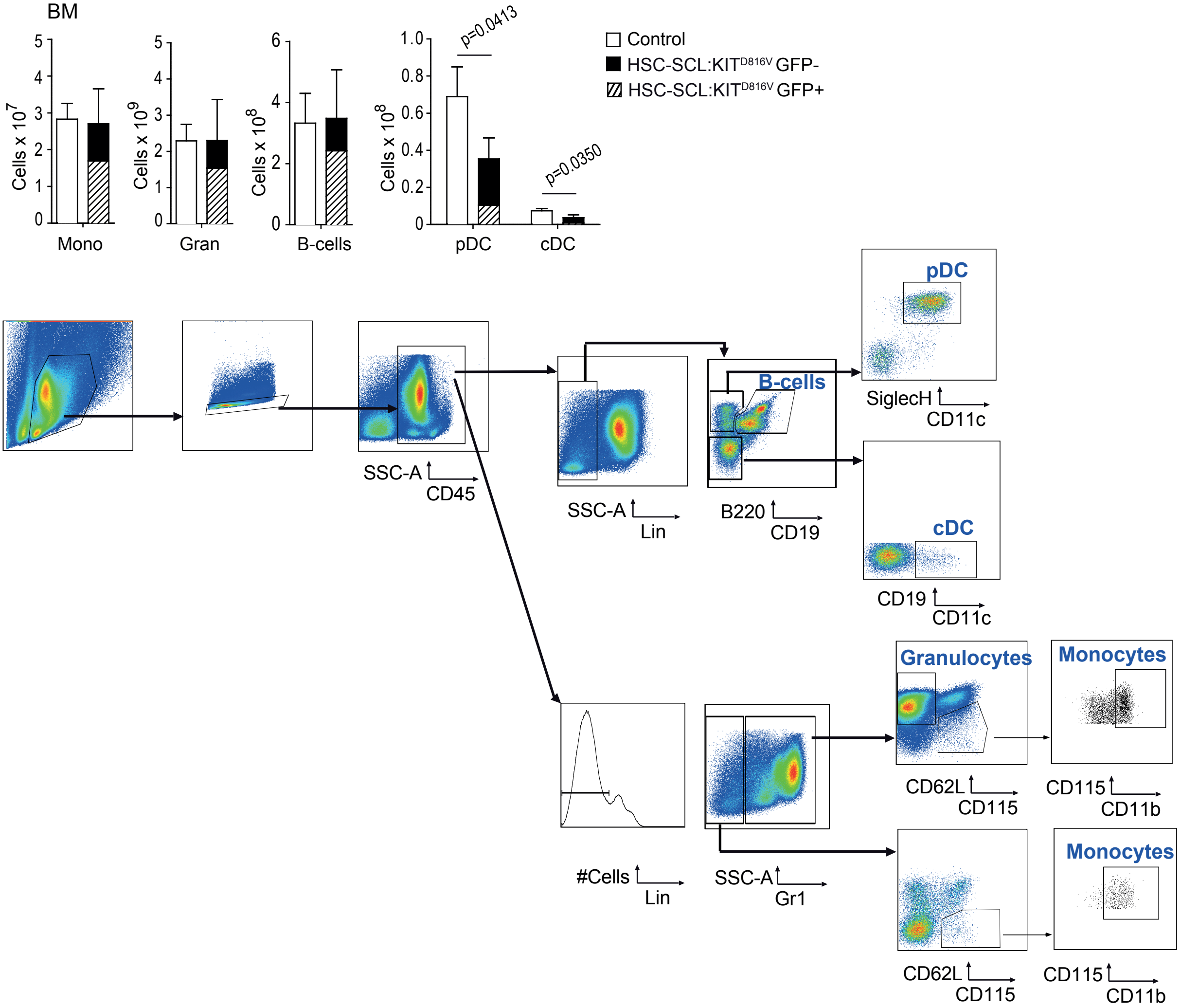
BM
Cells x 10^7
Cells x 10^9
Cells x 10^8
Cells x 10^8
Mono
Gran
B-cells
pDC
cDC
p=0.0413
p=0.0350
Control
HSC-SCL:KIT D816V GFP-
HSC-SCL:KIT D816V GFP+
SSC-A
CD45
SSC-A
Lin
B220
CD19
B-cells
pDC
SiglecH
CD11c
cDC
CD19
CD11c
#Cells
Lin
SSC-A
Gr1
Granulocytes
CD62L
CD115
Monocytes
CD115
CD11b
CD62L
CD115
Monocytes
CD115
CD11b

b

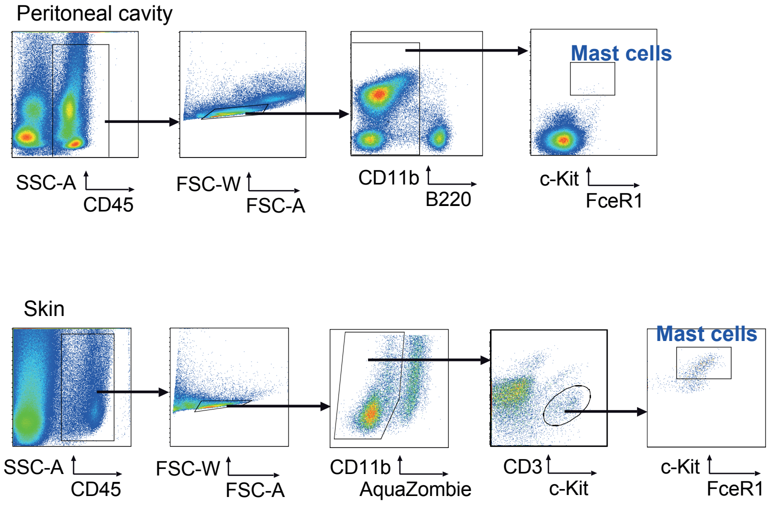
Peritoneal cavity
SSC-A
CD45
FSC-W
FSC-A
CD11b
B220
Mast cells
c-Kit
FceR1
Skin
SSC-A
CD45
FSC-W
FSC-A
CD11b
AquaZombie
CD3
c-Kit
Mast cells
c-Kit
FceR1

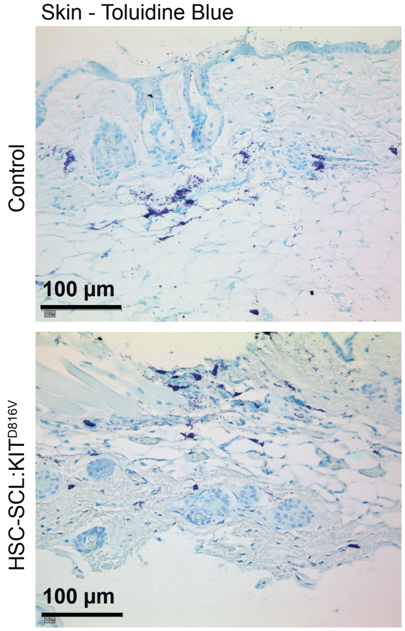
Skin - Toluidine Blue
Control
100 µm
HSC-SCL:KIT D816V
100 µm

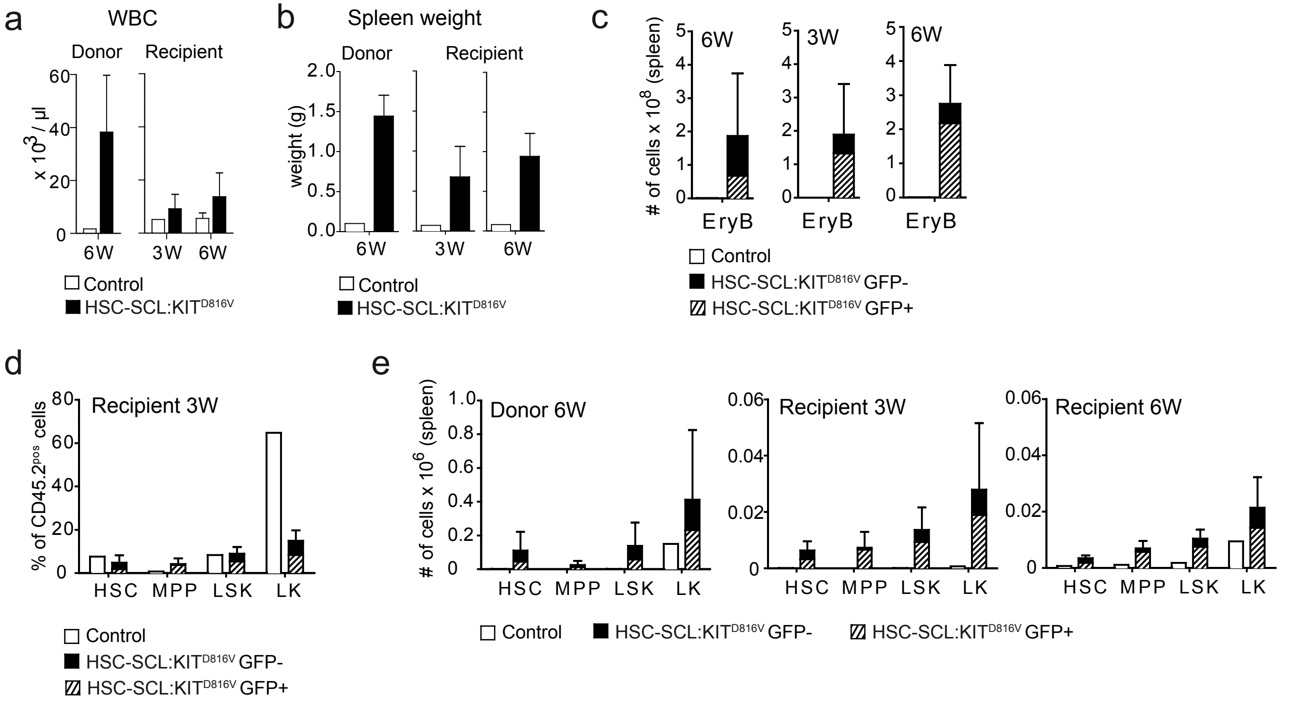
a
WBC
Donor
Recipient
x 10^3 / µl
60
40
20
0
6W
3W
6W
Control
HSC-SCL:KIT D816V
b
Spleen weight
Donor
Recipient
weight (g)
2.0
1.5
1.0
0.5
0.0
6W
3W
6W
Control
HSC-SCL:KIT D816V
c
# of cells x 10^8 (spleen)
6W
3W
6W
5
4
3
2
1
0
EryB
EryB
EryB
Control
HSC-SCL:KIT D816V GFP-
HSC-SCL:KIT D816V GFP+
d
Recipient 3W
% of CD45.2pos cells
80
60
40
20
0
HSC
MPP
LSK
LK
Control
HSC-SCL:KIT D816V GFP-
HSC-SCL:KIT D816V GFP+
e
# of cells x 10^6 (spleen)
Donor 6W
1.0
0.8
0.6
0.4
0.2
0
Recipient 3W
0.06
0.04
0.02
0
Recipient 6W
0.06
0.04
0.02
0
HSC
MPP
LSK
LK
Control
HSC-SCL:KIT D816V GFP-
HSC-SCL:KIT D816V GFP+

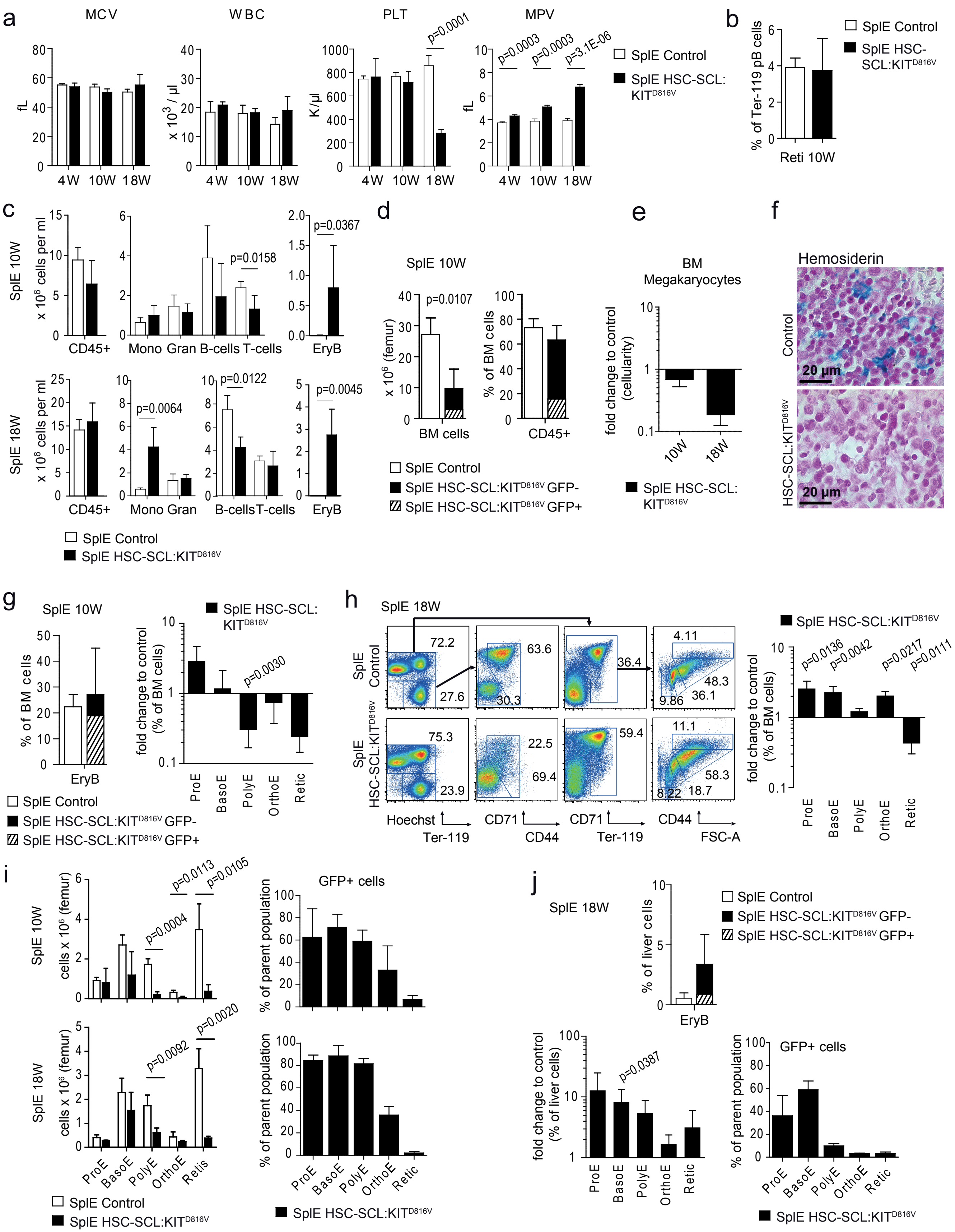
a
MCV
WBC
PLT
MPV
fL
x 10^3 / µl
K/µl
4W 10W 18W
p=0.0001
p=0.0003
p=0.0003
p=3.1E-06
SplE Control
SplE HSC-SCL:KIT^D816V
b
% of Ter-119 pB cells
Reti 10W
c
SplE 10W
SplE 18W
x 10^6 cells per ml
CD45+
Mono Gran B-cells T-cells
EryB
p=0.0158
p=0.0367
p=0.0064
p=0.0122
p=0.0045
SplE Control
SplE HSC-SCL:KIT^D816V
d
SplE 10W
x 10^6 (femur)
% of BM cells
BM cells
CD45+
p=0.0107
SplE Control
SplE HSC-SCL:KIT^D816V GFP-
SplE HSC-SCL:KIT^D816V GFP+
e
BM
Megakaryocytes
fold change to control
(cellularity)
10W
18W
SplE HSC-SCL:KIT^D816V
f
Hemosiderin
Control
HSC-SCL:KIT^D816V
20 µm
20 µm
g
SplE 10W
% of BM cells
EryB
fold change to control
(% of BM cells)
ProE BasoE PolyE OrthoE Retic
p=0.0030
SplE Control
SplE HSC-SCL:KIT^D816V GFP-
SplE HSC-SCL:KIT^D816V GFP+
h
SplE 18W
SplE Control
SplE HSC-SCL:KIT^D816V
72.2
27.6
63.6
30.3
36.4
4.11
48.3
9.86
36.1
75.3
23.9
22.5
69.4
59.4
11.1
58.3
8.22
18.7
Hoechst
Ter-119
CD71
CD44
CD71
Ter-119
CD44
FSC-A
SplE HSC-SCL:KIT^D816V
fold change to control
(% of BM cells)
p=0.0136
p=0.0042
p=0.0217
p=0.0111
ProE BasoE PolyE OrthoE Retic
i
SplE 10W
SplE 18W
cells x 10^6 (femur)
p=0.0004
p=0.0113
p=0.0105
p=0.0092
p=0.0020
ProE BasoE PolyE OrthoE Retis
SplE Control
SplE HSC-SCL:KIT^D816V
GFP+ cells
% of parent population
ProE BasoE PolyE OrthoE Retic
SplE HSC-SCL:KIT^D816V
j
SplE 18W
% of liver cells
EryB
SplE Control
SplE HSC-SCL:KIT^D816V GFP-
SplE HSC-SCL:KIT^D816V GFP+
fold change to control
(% of liver cells)
p=0.0387
ProE BasoE PolyE OrthoE Retic
GFP+ cells
% of parent population
ProE BasoE PolyE OrthoE Retic
SplE HSC-SCL:KIT^D816V

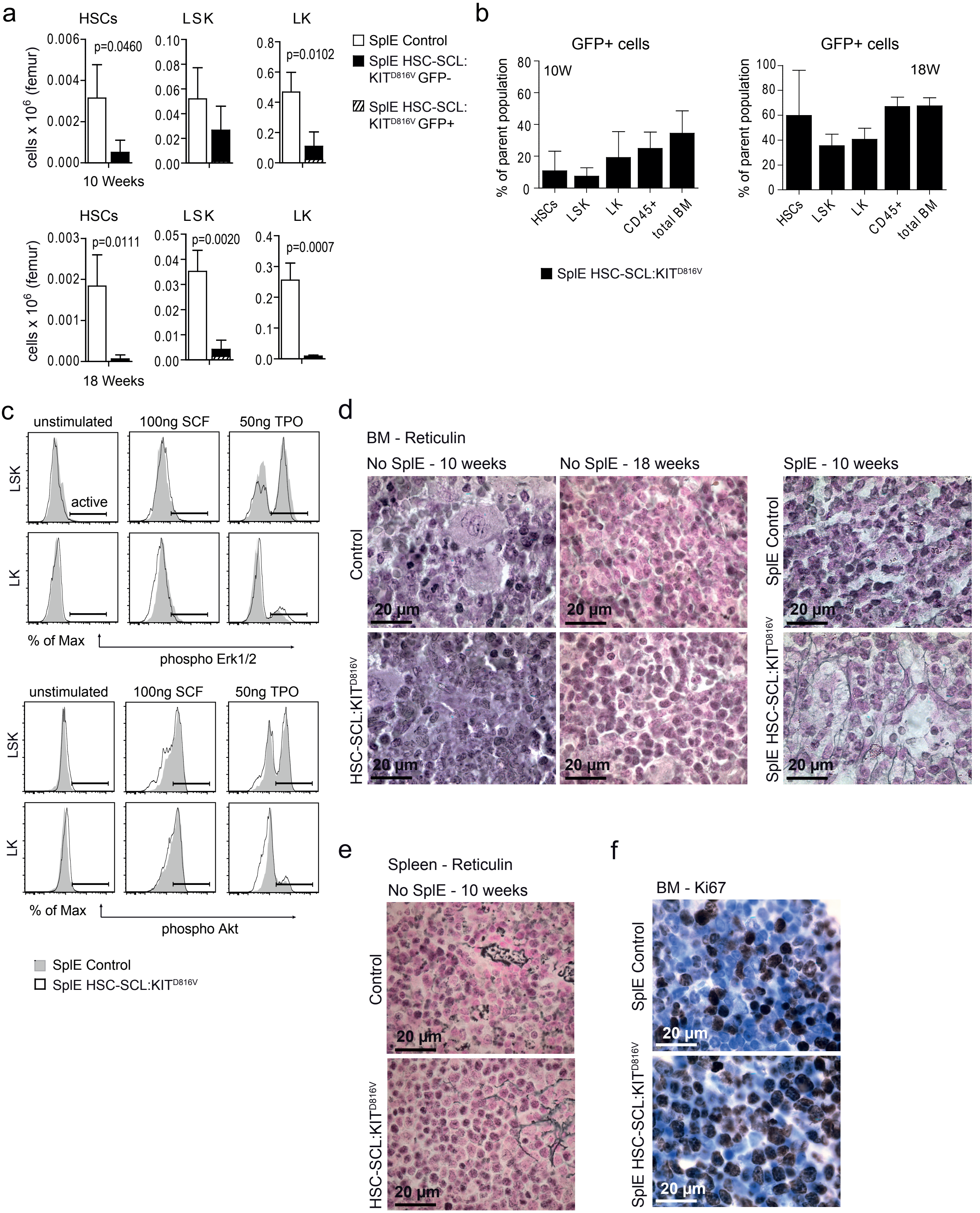
a
HSCs
LSK
LK
p=0.0460
p=0.0102
cells x 10^6 (femur)
10 Weeks
p=0.0111
p=0.0020
p=0.0007
18 Weeks
SplE Control
SplE HSC-SCL:KIT^{D816V} GFP-
SplE HSC-SCL:KIT^{D816V} GFP+
b
GFP+ cells
10W
18W
% of parent population
HSCs
LSK
LK
CD45+
total BM
SplE HSC-SCL:KIT^{D816V}
c
unstimulated
100ng SCF
50ng TPO
LSK
LK
active
% of Max
phospho Erk1/2
phospho Akt
SplE Control
SplE HSC-SCL:KIT^{D816V}
d
BM - Reticulin
No SplE - 10 weeks
No SplE - 18 weeks
SplE - 10 weeks
Control
HSC-SCL:KIT^{D816V}
SplE Control
SplE HSC-SCL:KIT^{D816V}
20 µm
e
Spleen - Reticulin
No SplE - 10 weeks
Control
HSC-SCL:KIT^{D816V}
20 µm
f
BM - Ki67
SplE Control
SplE HSC-SCL:KIT^{D816V}
20 µm
